# Type I interferons in bacterial diseases: myeloid cells at the crossroads of protection and pathology

**DOI:** 10.3389/fimmu.2025.1717370

**Published:** 2026-01-27

**Authors:** Irina Lyadova

**Affiliations:** Laboratory of Cellular and Molecular Basis of Histogenesis, Koltzov Institute of Developmental Biology of the Russian Academy of Sciences, Moscow, Russia

**Keywords:** bacterial infections, inflammatory hematopoiesis, macrophages, neutrophils, type I interferons

## Abstract

Type I interferons (IFN-I) are multifunctional cytokines with well-established antiviral and antitumor activities. In viral infections and cancer, IFN-I are largely protective, acting through both direct mechanisms, such as induction of antiviral or antiproliferative programs, and indirect mechanisms, mediated through the activation of immune effector cells. During bacterial infections, IFN-I primarily act indirectly, making their role more complex and contradictory. Depending on the context, IFN-I may promote host protection or contribute to pathology, and factors determining these divergent outcomes remain poorly understood. Comparative analysis of existing studies indicates that discrepancies in IFN-I effects arise from multiple pathogen- and host-dependent factors, including pathogen biology, the route of pathogen delivery, infection stage, host immune competence, the magnitude of IFN-I response and other parameters. Among them, the ability of IFN-I to reprogram myeloid cell responses appears to be a critical but insufficiently characterized determinant. This review synthesizes current evidence on IFN-I responses in bacterial infections, with particular emphasis on their effects in the myeloid cell compartment. These include IFN-I ability to inhibit macrophage activation, alter macrophage metabolism, induce myeloid cell death, affect macrophage and neutrophil recruitment, and modulate myeloid cell generation by supporting emergency hematopoiesis and redirecting lineage output toward monocyte or granulocyte generation. Given that macrophages and neutrophils differentially contribute to protection or pathology across various bacterial infections, such effects may underlie both beneficial and detrimental outcomes of IFN-I signaling. The review highlights IFN-I-driven regulation of myeloid cell activity and myelopoiesis as overlooked checkpoints in bacterial pathogenesis, providing a framework for future mechanistic studies and guiding the search for new opportunities in therapeutic intervention.

## 1 Introduction

Type I interferons (IFN-I) are pleiotropic cytokines implicated in both protection against and pathogenesis of infectious and oncological diseases. In viral infections and cancer, the protective effects of IFN-I generally predominate (1–4). Their antiviral activity is mediated through two principle mechanisms: direct induction of antiviral programs and indirect immune cell-mediated effects (2, 3, 5, 6). Similarly, in cancer, IFN-I act both directly on tumor cells by suppressing their proliferation and indirectly by promoting antitumor immune responses (7–9). In contrast, during bacterial infections, the effects of IFN-I are more complex and less predictable: they can be either protective or deleterious, and factors determining these outcomes remain poorly understood (10–12). A likely explanation is that IFN-I generally lack direct antibacterial activity and act primarily by modulating host immune reactions. This highlights the importance of unraveling the immunological consequences of IFN-I responses in bacterial infections. In this review, we examine the protective and pathological effects of IFN-I in selected bacterial infections and discuss the underlying immunological mechanisms, with emphasis on myeloid cells and IFN-I-induced myelopoietic shifts, two aspects of IFN-I activity that remain underappreciated.

## 2 IFN-I, IFN-I receptor and related signaling pathways

**Figure 1:**
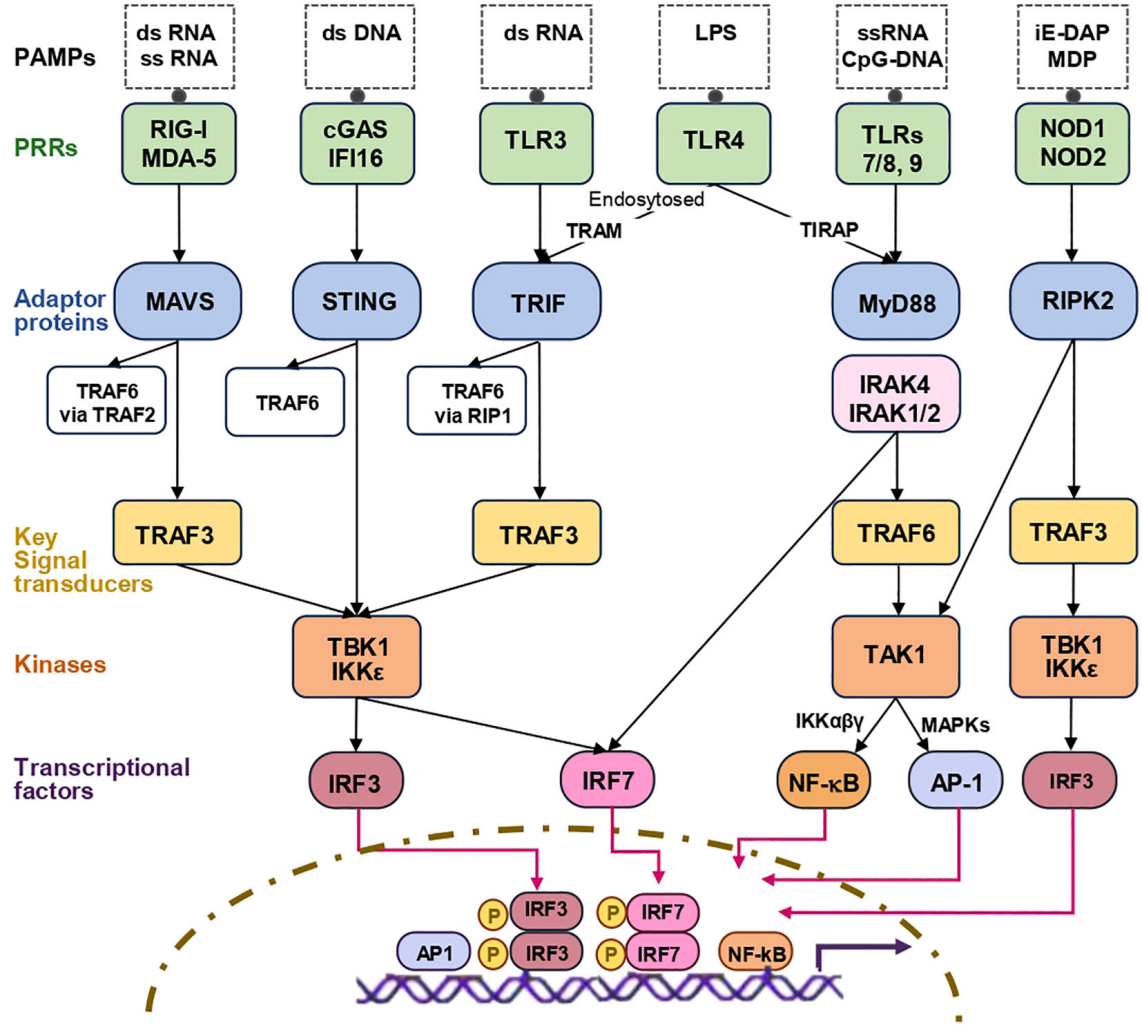
Schematic representation of the main signaling pathways leading to IFN–I induction. Pathogen–associated molecular patterns (PAMPs) trigger IFN–I production through various pattern recognition receptors (PRRs). *The cytosolic RNA sensors* RIG–I and MDA5 sense short dsRNA with 5’–triphosphate and long dsRNA, respectively. Upon RNA binding, RIG–I and MDA5 interact with the adaptor protein MAVS, located on the outer mitochondrial membrane. Activated MAVS recruits TRAF3, which in turn recruits and activates TBK1 and IKKε. TBK1 and IKKε phosphorylate IRF3 and IRF7 (in most cells, except pDCs, IRF7 must first be transcribed, a process induced by IFN–I). Phosphorylated IRF3 and IRF7 form homodimers, translocate to the nucleus and bind to the promoters of IFN–I genes to initiate transcription. MAVS also recruits TRAF2 and TRAF6, which stimulate: (i) the canonical IKK complex (IKKα, IKKβ, and NEMO/IKKγ) leading to the activation of NF–κB, and (ii) MAPKs leading to the activation of AP–1. NF–κB and AP–1 induce transcription of pro–inflammatory cytokines and enhance IFN–I transcription (which mainly depends on IRF3 and IRF7). At the scheme, only the main TRAF3–dependent pathway is illustrated. *cGAS*, *the main cytosolic DNA sensor*, recognizes dsDNA and catalyzes the synthesis of cyclic GMP–AMP (cGAMP). cGAMP binds to the adaptor protein STING, an ER transmembrane protein. STING translocates to the Goldgi compartment, where it oligomerizes and recruits TBK1. TBK1 phosphorylates STING. Phosphorylated STING recruits IRF3, which is phosphorylated by TBK1. STING can also recruit TRAF6, leading to activation of NF–κB and AP–1 (not depicted in the scheme). *The endosomal RNA sensor TLR3* recognizes long dsRNA, binding induces TLR3 dimerization, which results in the recruitment of the adaptor protein TRIF. TRIF recruits TRAF3, which activates the TBK1/IKKε–IRF3/IRF7 pathway. TRIF can also recruit RIP1 and TRAF6, which leads to the activation of NF–κB and AP–1. The e*ndosomal receptors TLR7 and TLR8 recognize ssRNA, and TLR9* recognizes unmethylated CpG DNA. Upon binding their ligands, these TLRs recruit MyD88 via their TIR domains. MyD88 oligomerizes and recruits IRAK4, which phosphorylates IRAK1 and IRAK2, forming the Myddosome complex. Activated IRAK1/2 recruit TRAF6, which together with other proteins recruits TAK1. TAK1 activates the IKK complex, resulting in: (i) NF–κB activation and nuclear translocation, and (ii) MAPK activation (p38, ERK, JNK), which activates AP–1. The MyD88➔IRAK4➔IRAK1 pathway also recruits and activates IRF7, particularly in plasmocytoid DCs. *The membrane receptors TLR2 and TLR4* recognize cell wall components of gram–positive and gram–negative bacteria, respectively. Ligand binding results in the activation of NF–kB and AP–1 via MyD88–IRAK signaling cascade. TLR4 can be endocytosed and directed to endosomes, where it recruits TRAM and TRIF, leading to the sequential activation of TRAF3, TBK1/IKKε, IRF3 and IRF7, as described above. TRIF can also activate NF–κB and AP–1 via RIP1–TRAF6 pathway. *NLRs are cytosolic sensors of PAMPs/DAMPs*. Upon recognizing their ligands, muramyl dipeptide and γ–D–glutamyl–meso–diaminopimelic acid, NLRs self–oligomerize and recruit RIPK2, which subsequently recruits TAK1 and the IKK complex leading to the activation of NF–kB. RIPK2 also recruits TRAF3, which activates the TBK1➔IRF3/IRF7 cascade. In some cell types, such as DCs and macrophages, NLRs may also trigger IRF3 through interactions with MAVS or TRAF3. More detailed information on IFN–I–inducing signaling pathways can be found in comprehensive reviews (23–28). AP–1, Activating protein–1; cGAS, Cyclic GMP–AMP synthase; iE–DAP, γ–D–glutamyl–meso–diaminopimelic acid; IKK, I Kappa B Kinase; IRAK, Interleukin–1 receptor associated kinase; IRF3, Interferon Regulatory Factor 3; IRF7, Interferon Regulatory Factor 7; LPS, Lipopolysaccharide; TLR, toll–like receptor; LTA, lipoteichoic acid; MAPKs, Mitogen–activated protein kinase; MAVS, Mitochondrial antiviral–signaling protein; MDA–5, Melanoma Differentiation–Associated protein 5; MDP, muramyl dipeptide; MyD88, Myeloid differentiation primary response 88; NF–kB, Nuclear factor kappa–light–chain–enhancer of activated B cells; NLRs, nucleotide oligomerization domain receptors; PAMP, pathogen–associated molecular patterns; PGN, peptidoglycan; PRRs, pattern recognition receptors; RIG–1, retinoic acid–inducible gene 1; RIP1, serine/threonine kinase receptor–interacting protein 1; RIPK2, Receptor–interacting serine/threonine–protein kinase 2; STING, Stimulator of interferon genes; TAK1, TGFβ activated kinase 1; TBK–1, TANK–binding kinase 1; TIRAP, toll–interleukin 1 receptor (TIR) domain containing adaptor protein; TRAF3, TNF receptor–associated factor 3; TRAF6, TNF receptor–associated factor 6; TRAM, TRIF–related adaptor molecule; TRIF, TIR (Toll/interleukin–1 receptor) domain–containing adaptor protein.

IFN-I are conserved cytokines consisting of IFN-α, IFN-β, IFN-ϵ, -κ, –ω, -δ, -τ, and ζ of which IFN-α and IFN-β are most abundantly produced and best-studied. In humans, IFN-α has 12 different isoforms (14 in mice) encoded by 14 genes (one of which is a pseudogene, and two of which encode similar proteins); IFN-β is encoded by only one gene (13). IFN-α are produced predominantly by immune/hematopoietic cells, whereas IFN-β is produced by most cells in the body (14, 15). In homeostatic conditions, IFN-I are expressed at low levels. Their expression rapidly increases in response to unusual nucleic acids and the components of bacteria cell wall that act as ligands for host cell pattern recognition receptors (PRRs). The PRRs that trigger IFN-I response include: (i) surface toll-like receptors TLR2 and TLR4 (16, 17), (ii) endosomal receptors TLR3, TLR7/8 and TLR9 (18), (iii) cytosolic RNA sensors Retinoic acid–inducible gene I (RIG–I) and Melanoma differentiation–associated gene 5 (MDA–5) (19), (iv) cytosolic DNA sensors Cyclic GMP–AMP synthase (cGAS), Interferon–γ Inducible Protein 16 (IFI16), DNA–dependent activator of IFN–regulatory factors (DAI), Absent in melanoma 2 (AIM2), and DEAD box polypeptide 41 (DDX41) (20, 21), and (v) Nucleotide–binding oligomerization domain–containing proteins (NOD1/NOD2) (22). These PRRs recognize various ligands, and together, enable cells to respond to a broad range of foreign pathogens and self–derived damage signals by producing IFN–I (summarized in **Figure 1**). Signaling pathways linking PRRs with the induction of IFN–α/IFN–β have been reviewed elsewhere (23, 24), and are briefly summarized here to the extent necessary for the current review. *IFNB* and *IFNA* genes are induced via similar, yet distinct, pathways. The promoter of the *IFNB* gene contains four positive regulatory domains (PRDI–IV) that bind transcriptional factors Interferon regulatory factor 3 (IRF3), IRF7, Nuclear factor kappa–light–chain–enhancer of activated B cells (NF–κB) and Activator protein 1 (AP–1). The induction of *IFNB* transcription requires the assembly of several transcriptional complexes at its promoter (29–32). The induction of *IFNA* does not directly depend on NF–κB or AP–1 and can be induced only by IRFs, such as IRF1, IRF3, IRF4, IRF5, IRF7 and IRF8 (30). Among IRFs, IRF3 and IRF7 are central for the induction of IFN–I (25, 33). To bind the *IFNB* promoter, IRF3 must be phosphorylated, form homodimers and translocate to the nucleus. Kinases responsible for IRF3 phosphorylation are TANK–binding kinase 1 (TBK1) and the inhibitor of κB kinase–related kinase–ϵ (IKK–ϵ). Both kinases are activated in response to the ligation of TLR3 and intracellular RNA and DNA sensors. (**Figure 1**, see the legend to **Figure 1** for more detailed information) (23, 32, 34). TBK1/IKKε also phosphorylate IRF7. However, while IRF3 is constitutively expressed in most cells and can be phosphorylated rapidly, IRF7 starts to be expressed only in response to IFN–α/IFN–β. Therefore, IRF7 joins the response later than IRF3 (35, 36). An exception is plasmocytoid dendritic cells (pDCs) in which IRF7 is expressed constitutively and supports early production of IFN–α (37). Endosomal TLRs 7/8 and TLR9 through the adaptor proteins Myeloid differentiation primary response 88 (MyD88) and TRAF6 activate another kinase, Transforming Growth Factor Beta Activated Kinase 1 (TAK1), which leads to the activation of NF–κB and AP–1 (38). MyD88–dependent pathway also activates IRF7, particularly, in plasmocytoid DCs (18). TLR4 induces MyD88–dependent activation of NF–kB and MyD88–independent activation of IRF3 (18, 34) (**Figure 1**). Nod‐like receptors (NLRs) induce the activation of NF–kB and IRF3 through the receptor–interacting serine/threonine–protein kinase 2 (RIPK2)– dependent cascades (25–28) (**Figure 1**). In different cells, different pathways of IFN–I induction predominate (37, 39, 40).

**Figure 2:**
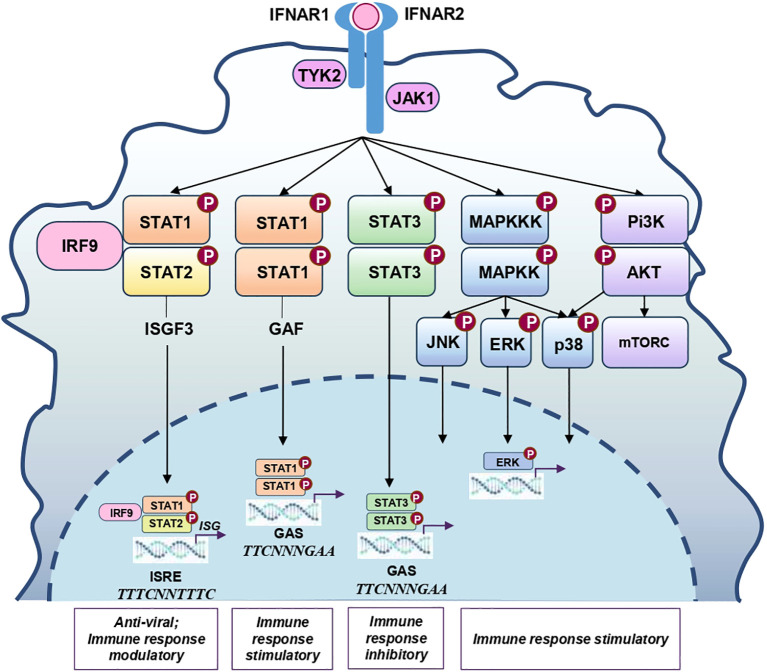
Schematic representation of the main signaling pathways induced by IFN–I. Binding of IFN–α/β to the IFNAR activates receptor–associated kinases JAK1 and TYK2. The kinases phosphorylate STAT1, STAT2, STAT3, as well as several other proteins. Phosphorylated STAT1 and STAT2 form STAT1–STAT2 heterodimers that associate with IRF9 constitutively expressed within a cell, to form the ISGF3 complex. ISGF3 translocates to the nucleus and binds ISRE in the promoters of the target genes. IRF9 determines the binding specificity of the ISGF3, STAT1 and STAT2 recruit transcriptional coactivators, such as CBP/p300. Phosphorylated STAT1 form homodimers that translocate to the nucleus, bind GAS elements and induce the transcription of immune response genes, such as IRF1, MHC class II, SOCS1 etc. Phosphorylated STAT3 form homodimers that also translocate to the nucleus and bind GAS elements. Differentially form STAT1–STAT1 homodimers, STAT3–STAT3 homodimers recruit a different set of cofactors, resulting in the induction of a different set of genes, primarily those related to cell survival, proliferation and immune modulation (e.g., IL–10), VEGF and c–Myc). Ligation of IFNAR also induces the recruitment of certain adaptor proteins, such as Shc and Grb2. Some adaptor proteins activate MAPKKKs initiating the cascade that leads to the phosphorylation of MAPKs ERK, p38 and JNK and the subsequent induction of genes involved in early immune response, cell proliferation, differentiation and survival. Other adaptor proteins activate PI3K leading to the activation of AKT and mTORC1 and promoting cell survival, protein synthesis and cell metabolic reprogramming. AKT, Protein Kinase B; CBP, CREB–binding protein; c–Myc, cellular myelocytomatosis oncogene; ERK, Extracellular signal–regulated kinase; GAF, γ–activated factor; GAS, γ–activated sequence; IRF, Interferon regulatory factor; ISGF3, Interferon–stimulated gene factor 3; ISRE, IFN–stimulated response elements; JAK1, Janus kinase 1; JNK, c–Jun N–terminal kinase; MAPKK, Mitogen–activated protein kinase kinase; MAPKKK, Mitogen–activated protein kinase kinase kinase; MHC, major histocompatibility complex; mTORC, Mammalian Target of Rapamycin; P38, p38 kinase; Pi3K, Phosphatidylinositol 3–kinase; SOCS, suppressor of cytokine signaling proteins; STAT, Signal transducer and activator of transcription 1; TYK2, Tyrosine kinase 2; VEGF, Vascular Endothelial Growth Factor.

Once produced, IFN–I bind to the IFNAR receptor. IFNAR is a heterodimer receptor composed of IFNAR1 and IFNAR2 chains that are ubiquitously expressed on different cells and tissues, although at different levels (41). The ligation of IFNAR induces canonical and non–canonical signaling pathways (**Figure 2**). The canonical signaling cascade leads to the formation of STAT1–STAT2 heterodimers, the binding of IRF9 to STAT1/STAT2 heterodimers, the formation of the three–molecular complex Interferon Stimulated Gene Factor 3 (ISGF3), and the translocation of the ISGF3 to the nucleus, where it binds to interferon–stimulated response elements (ISREs) and induces the expression of interferon–stimulated genes (ISGs). Some ISGs (e.g., *EIF2AK2, MX1, OAS1, ISG15* etc.) exert direct antiviral activity by inhibiting viral entry, primary transcription, translation and replication (reviewed in detail in (5, 8, 42, 43)). Other ISGs stimulate the death of infected cells and/or modulate the activity of innate and adaptive immune cells by inducing NF–kB, MAPK and isgylation of target proteins (reviewed in (2, 42, 43)). The presence of ISREs in the promoter regions of the *IFNB* and *IFNA* underlies the autocrine and paracrine regulation of IFN–α/IFN–β production.

In addition to inducing STAT1/STAT2 heterodimers, IFNAR engagement also promotes the formation of other STAT dimers, including STAT1/STAT3 heterodimers, as well as STAT1, STAT3, STAT4, STAT5 and STAT6 homodimers (44–46). The STAT1/STAT1 homodimers bind to the interferon–gamma–activated sequence (GAS) and activate immune–stimulatory genes (e.g., IRF1). STAT3/STAT3 homodimers also bind GAS, but act primarily by negatively fine–tuning the response (46). Non–canonical pathways activate MAPK kinases and the phosphoinositide 3–kinase (PI3K)/mammalian target of rapamycin (mTOR) pathways that influence ISG transcription either independently of, or in conjunction with, the canonical JAK–STAT pathway (44, 45, 47).

## 3 Bacterial infections induce IFN–I

Bacterial infections are potent inducers of the IFN–I response. The induction of IFN–I has been observed in *in vivo* and *in vitro* models following the infection with gram–negative, gram–positive, intracellular and extracellular bacteria. Some examples are provided below (see **Box 1** for the main characteristics of considered pathogens).

Box 1 **Main characteristics of considered bacterial pathogens.**

*Listeria monocytogenes* (LM)
Gram–positive, facultative intracellular motile rod–shaped foodborne pathogen, causes listeriosis, which ranges from mild gastrointestinal disease to severe infections, including meningitis and septicemia, primarily affecting newborns, the elderly, and immunocompromised individuals. Can present as both acute and chronic infection. Within infected host cells, LM rapidly escapes from phagosomes into the cytosol.
*Salmonella enterica* serovar  Typhimurium (ST)
Gram–negative intracellular motile rod bacterium. ST causes gastroenteritis and enterocolitis, whereas serovars Typhi and Paratyphi cause systemic typhoid and paratyphoid fever. Within infected cells, ST primarily resides in phagosome–derived Salmonella–containing vacuoles (SCVs), limited cytosolic escape has also been reported.
*Francisella tularensis* (FT)
Gram–negative, facultative intracellular non–motile coccobacillus. Causes tularemia, an acute febrile zoonotic infection. Disease can be triggered by very small infectious doses via multiple routes (inhalation, skin contact or mucosal exposure) and has various clinical forms, of which the respiratory form is the most life–threatening. In host cells, FT rapidly escapes from phagosomes into the cytosol, where it replicates and can persist for prolonged periods.
*Mycobacterium tuberculosis (Mtb)*
Acid–fast, facultative intracellular non–motile rod, slow–growing pathogen. Causes pulmonary or extrapulmonary tuberculosis or latent infection. *Mtb* primarily persists within phagosomes by inhibiting phagolysosomal maturation, but can disrupt phagosomal membrane and access the cytosol, the process that contributes to bacterial replication.
*Pseudomonas aeruginosa* (PA)
Gram–negative, extracellular motile rod bacterium, an opportunistic pathogen. Causes pneumonia, folliculitis, otitis, osteomyelitis, sepsis, and various nosocomial infections. Infections are common in immunocompromised individuals and can be acute or chronic. PA does not persist intracellularly under most conditions.
*Klebsiella pneumoniae* (KP)
Gram–negative, extracellular non–motile rod pathogen. Causes pneumonia, urinary tract infections, sepsis and meningitis. KP does not persist in phagosomes or the cytosol.
*Streptococcus pneumoniae* (SPn) and *Streptococcus pyogenes* (Spy)
Gram–positive extracellular non–motile cocci. SPn causes pneumonia, meningitis, otitis and sepsis, SPy causes pharyngitis, skin and soft–tissue infections, and invasive diseases such as necrotizing fasciitis. Both pathogens are predominantly extracellular and do not persist in phagosomes or the cytosol.
*Staphylococcus aureus* (SA)
Gram–positive non–motile cocci, opportunistic pathogen causing various diseases ranging from minor skin infections to severe illnesses, such as life–threatening pneumonia, abscesses, osteomyelitis and sepsis. SA was initially classified as extracellular pathogen, but later shown to invade various host cells. Inside the cells, SA may persist within phagosomes and, in some cases, escape into the cytosol. Includes methicillin–resistant SA (MRSA) strains.

### 3.1 IFN–I response during intracellular bacterial infections

*Listeria monocytogenes* (LM) induces a robust production of IFN–β. The response has been observed in *in vivo* mouse models, where cells of monocyte/macrophage lineage, including monocyte–derived dendritic cells producing TNF–α and nitric oxide (Tip–DCs) were the main source of IFN–I (48–54) In *in vitro* systems, LM stimulated IFN–β expression in mouse and human macrophages, monocyte–like cell lines, mouse embryonic fibroblasts and HELA cells (55–58). Once infecting the target cell, LM rapidly escapes form the phagosome. The escape is a prerequisite for the induction of IFN–β, which depends primarily on the engagement of cytosolic DNA sensors and the activation of the STING➔TBK1➔IRF3 pathway (53, 55, 57–62). Additionally, TLR2, TLR3 and the activation of NF–κB and p38 Mitogen–activated protein kinase (MAPK) have been implicated in IFN–β induction (55–57).

*Salmonella* primarily invades intestinal epithelial cells but can also infect other cell types and disseminate to distal organs (63). Salmonella–induced expression of IFN–β has been observed in mouse and zebrafish *in vivo* models, as well as in mouse embryonic fibroblasts, epithelial cells and mouse and human macrophages infected *in vitro* (64–67). Intracellularly, *Salmonella* persists within a phagosome–derived membrane compartment, the *Salmonella*–containing vacuole (SCV) for extended periods, it and its DNA can leak into the cytosol upon SCV membrane damage (68, 69). Consequently, IFN–I induction occurs through multiple pathways, including endosomal TLR4– and TLR3–TRIF signaling, as well as STING triggered by cytosolic *Salmonella* DNA or by mitochondrial DNA released during infection–associated mitochondrial stress (67, 70–73).

*Francisella tularensis* causes tularemia and is highly virulent, therefore, most studies have been performed with less virulent strains, such as *F. novicida* and the *F. tularemia* live vaccine strain (LVS). *Francisella* persists primarily in macrophages, but can also invade other phagocytic (DCs, neutrophils) and non–phagocytic (B–lymphocytes, endothelial and epithelial cells, and hepatocytes) cell types (74). Due to its atypical LPS, *Francisella* is poorly recognized by TLR4 but can activate surface and endosomal TLR2, which mediates the initial IFN–I response (75–77). Following phagocytosis, *Francisella* rapidly escapes into the cytosol, where its nucleic acids are sensed by STING, RIG–I and AIM2 (72, 77–83). This cytosolic recognition is essential for full IFN–β induction; secreted IFN–β can further promote pathogen sensing, including via the induction of AIM2 (80). Notably, human monocytes show a lower response to *F. novicida* compared with the virulent *F. tularensis* SCHU4 strain, and AIM2 expression is lower in human monocytes than in murine cells (82, 84, 85). Thus, not all mechanisms established in experimental models mirror those occurring during *F. tularensis* infection in humans.

Escaping from phagolysosome is also necessary for the effective induction of IFN–I by mycobacteria. In the context of mycobacterial infections, IFN–I response has been registered in peripheral blood cells of tuberculosis (TB) patients (86), in the serum and lung cells of infected mice (87–89) and in mouse and human macrophages challenged *in vitro* (88, 90–92). The primary virulence factor of *Mycobacterium tuberculosis* (*Mtb*), the ESX–1 secretion system, mediates *Mtb* escape from the phagosome (93) and is a prerequisite for IFN–I induction (94). The main involved signaling pathways are cGAS– and RIG–1–mediated (91, 92, 94). In addition, cyclic diadenosine monophosphate (c–di–AMP) released by mycobacteria can directly bind to STING or to DDX41 and activate STING–mediated signaling in a cGAS–independent way; *Mtb*–induced mitochondrial stress and a release of mitochondrial DNA contribute to IFN–I induction (95).

### 3.2 IFN–I response during extracellular bacterial infections

The induction of IFN–I by extracellular bacteria has been documented in *in vivo* and *in vitro* models of *Pseudomonas aeruginosa* (PA)*, Klebsiella pneumoniae* (KP), *Streptococcus pneumonia* (*SPn*), *Streptococcus pyogenes* (*SPy*) and other infections (96–101). Extracellular bacteria trigger IFN–I production by engaging surface PRRs, as well as by accessing intracellular PRRs through bacteria–dependent permeabilization of the host cell membrane or via the internalization of the ligated surface PRR (98, 102). PA primarily induces the IFN–I response through surface TLR4 (98, 100). In addition, TLR2 recognizes PA‐derived lipoproteins, TLR5 detects PA flagellin, and cGAS senses PA DNA (103). Similarly to PA, KP, activates the IFN–I response predominantly via TLR4 with additional contributions from TLR2 and cGAS (104, 105). The induction of IFN–I by SPn and SPy depends on cytolysins (pneumolysin and streptolysin, respectively), pore–forming virulence factors that enable bacterial material to enter the host cytosol and activate DNA and RNA sensors. SPn DNA can also be transcribed into a 5’–triphosphate RNA, which activates RIG–I (98, 99). IFN–I induction by SPy was shown to vary by host cell type: in macrophages, IFN–I response was mediated via the STING–IRF3 pathway, with only partial dependence on MyD88, whereas in conventional DCs (cDCs) the response was MyD88– and IRF5–dependent (39). The induction of IFN–I by extracellular bacteria via STING–dependent pathways is not surprising as many of them, including, PA, KP, SPn and SPy are able to penetrate cells; their products can also be liberated from phagosomes and subsequently detected by cytosolic PRRs (96).

### 3.3 IFN–I response to *Staphylococcus aureus*

*Staphylococcus aureus* (SA) was traditionally classified as an extracellular bacterium, but has later been shown to invade a variety of host cells, including professional phagocytes, endothelial and epithelial cells, fibroblasts, oesteoblasts, and keratinocytes (106–108). Therefore, SA is here considered separately from strictly intracellular and extracellular bacteria. To enter host cells, SA uses surface adhesins, such as fibronectin–binding proteins A and B, which engage host integrins and enable SA entry via a zipper–type mechanism (109). Once intracellular, SA initially resides in phagosomes/lysosomes, where it activates TLR9 (primarily in DCs; triggered by bacterial CpG DNA) and TLR8 (primarily in macrophages; induced by uridine–rich RNA) (110–112). Following phagosomal escape, SA can replicate in the cytosol, where it stimulates IFN–I production through NOD1/2 activation and the detection of c–di–AMP by STING (113). Extracellular SA triggers IFN–I primarily via TLR2 (112). Protein A has been shown to contribute to IFN–β production (114). Different SA stains can induce varying levels of IFN–I, even when localized in the same cellular compartment (101). The strength and pathways of IFN–I induction also depend on the mode of SA growth: biofilms have been shown to alter the STING–IRF3 pathway and *Ifnb* induction, whereas this was not observed in planktonic SA forms (115). The dual intra– and extracellular lifestyle of SA underlies its capacity to modulate innate immune signaling through multiple pathways and across diverse cell types.

Overall, bacteria are potent inducers of IFN–I; the prevailing signaling pathways depend on the pathogen, its localization and mode of growth, and the host cell type (8, 39). Comprehensive reviews detailing molecular pathways of IFN–I induction by bacteria are available elsewhere (12, 24, 33, 34, 72).

## 4 In the context of bacterial infections, IFN–I exert both protective and deleterious effects

Each infectious disease has its own characteristics. Nevertheless, in experimental conditions, the severity of most infections and the effectiveness of host protection against them can be evaluated based on similar criteria, which include host survival, pathogen loads and target tissue pathology. To determine how IFN–I affect these parameters, three main approaches have been used. The first approach implies the analysis of infection outcomes in mice or cells lacking IFN–I signaling – either as a result of anti–IFNAR1 treatment or due to genetic deletion of *Ifnar1* or down–stream signaling molecules. The second approach examines the influence of exogenous IFN–I or their inducers [e.g., polyinosinic:polycytidylic acid (poly(I:C)) or viruses] on the course of infection. The third approach studies the relationship between the magnitude of the IFN–I response and the severity of infectious diseases in humans. Despite multiple studies, there is currently no complete understanding on how IFN–I impact the pathogenesis of bacterial infections. The controversy of results obtained is illustrated below with examples of selected bacterial infections and a brief discussion of the main immunological findings.

### 4.1 Infection with *Listeria monocytogenes*

LM induces an acute infection, the protection depends on the production of IFN–γ secreted by γδ and NK cells early after infection and by Th1 and CD8^+^ lymphocytes at late stages (116). Numerous studies have shown that *Ifnar1*–deficient mice exhibit increased resistance to systemic LM infection induced by intravenous (i.v.) or intraperitoneal (i.p.) inoculation of the pathogen (54, 59, 62, 117–120). The enhanced resistance of *Ifnar1^–/–^* mice to LM has been linked to various immunological mechanisms, including: (i) elevated levels of IL-12p70 (117), (ii) improved generation of antigen–specific IFN–γ–producing CD8^+^ and T–memory cells (62, 121), (iii) enhanced macrophage responsiveness to IFN–γ (122), (iv) reduced production of IL–10 (54), (v) increased recruitment of protective TNF–α–producing CD11b^+^ cells (117) and neutrophils (123) to infection sites, (vi) reduced T–cell apoptosis and improved splenocyte survival (59) and (vii) reduced bacterial dissemination (119) (see **Table 1** for summarized data).

**Table 1:** The diversity of IFN–I–related effects during *Listeria monocytogenes* infection*.

Reference	Mice, background, Cells	Challenge and other experimental details	Conclusions on IFN–I effects on*:								
			Disease course	Th1 response, IFN–γ, T–cells	IL–1β	Proinflammatory cytokines/chemokines	IL–10	Mph/DC activation	Mph and DC recruitment	Neu recruitment	Immune cell viability
(59)	*Ifnar1^–/–^*, *MyD88^–/–^*, *Irf3^–/–^*, B6, BMDMs	LM: i.v. Poly(I:C), *in vitro*	D	NE: serum IFNγ	NE	NE: IL–6	NA	NA	NA	NA	↑ spleen cell apoptosis
(62)	*Gt, Ifnar1^–/–^*, *MyD88^–/–^*, *Irf3^–/–^*, *Irf3/7^–/–^*, B6	LM–WT, LM–ΔactA: i.v., i.v. + c–di–AMP	D	↓ IFN–γ^+^TNFα^+^, CD69^+^ T–cells	NA	↓ IL–6, TNF–α	NA	↓ CD86, CD40	NA	NA	NA
(117)	*Ifnar1^–/–^*, BALB/c, BMDMs, pMphs	LM: i.v.	D at late stages	NA	NA	↓ TNF–α, IL–12p70 ↑ IL–6, MCP–1	NA	NA	↓ TNF–a^+^CD11b^+^	NA	NA
(121)	*Ifnar1^–/–^*, *Caspase1^–/–^*, B6, BMDMs	Attenuated LM expressing OVA, i.v.	NA	↓ IFNγ^+^, IFNγ^+^TNFα^+^IL2^+^	NA	NA	NA	NA	↓ DC–dependent T–activation	NA	NA
(120)	*Ifnar1^–/–^*, *Padi4*^–/–^, B6	LM: i.v., intravital liver imaging	D	NA	NA	NA	NA	NA	NA	↓ of Neu swarming	NA
(54)	*Ifnar1^–/–^*, *Ifng^–/–^*, B6, BMDMs	LM: i.v.	D	↓ T–cells in spleen	NA	NA	↑ IL–10 (spleen)	NE: IFNGR expression	NA	↓ CD11b^+^ Ly6G^hi^Ly6C^int^	↑ T–cell death
		LM: f.b.	NE	NE	NA	NA	NE	NE: IFNGR	NA	NE	NE
(118)	*Ifnar1^–/–^*, B6, BMDMs	LM: i.p., i.v., *in vitro*	D	NA	NA	NA	NA	NA	NA	↑ Ly–6G^+^Ly6C^+^	↑TUNEL^+^ cells, spleen
		LM: i.g.	P	NA	NA	NA	NA	NA	↑ F4/80^+^ & Ly6C^+^Ly6G^–^	↑ Ly–6G^+^Ly6C^+^	↑TUNEL^+^ cells, spleen
(124)	*Ifnar1^–/–^*, *MyD88^–/–^*, *Ifnar1 ^–/–^MyD88^–/–^*, *MCP1*^–/–^, B6, BMDMs	LM: i.v, *in vitro*	P in MyD88^–/–^	NA	NA	↑ MCP–1	NA	NA	↑ Protective Ly–6C^hi^ in MyD88^–/–^ mice	NA	NA
(125)	WT B6, BMDCs	LM+IFN–β i.v., *in vitro* BMDCs+NK	P	↑ IFN–γ by NK cells	NA	NA	NA	NA	NA	NA	NA
(123)	*Ifnar1^–/–^*, B6 BMDCs	LM: i.p., *in vitro*	NA	NA	NA	↓ CXCL1, CXCL2, IL–12p40, CXCL4, CSF2, CSF3	NA	NA	NA	↓ CD11b^+^Gr–1^+^Ly–6C^int^	NA
(122)	*Ifnar1^–/–^*, *Il6*^–/–^, *Ifng^–/–^*, *Ifngr^–/–^*, B6, BMDMs, RAW264.7	LM: i.v, *in vitro*	NA	NA	NA	NA	NA	↓ Mph response to IFN–γ	NA	NA	NA

*Different studies assessed IFN–I effects under various conditions, such as loss of IFN–I signaling or its direct stimulation. For consistency, the table shows inferred IFN–I effects rather than raw data; for example, increased resistance of IFNAR^–/–^ mice is interpreted as a deleterious action of IFN–I; reduced function in IFNAR^–/–^ mice is interpreted as function being stimulated by IFN–I. Color code: Pink, function repressed by IFN–I; green, function enhanced by IFN–I; yellow, no effect observed; blue, opposite effects reported for different factors or in different conditions. Specific labels: ↑, increase, ↓, decrease/repression, D, detrimental, P, protective, NA, not analyzed, NE, no effect detected.

α–IFNAR1, antibodies against the IFN–α/β receptor; actA, LM virulence factor ActA; B6, C57Bl/6 mice; BMDCs, bone marrow–derived dendritic cells; BMDMs, bone marrow–derived macrophages; c–di–AMP, cyclic di–AMP; f.b., foodborn; Gt, Goldenticket mice lacking STING; i.g., intragastric; i.p., intraperitoneal; i.v., – intravenous; Neu, neutrophils; NK, natural killer cells; OVA, ovalbumin; Padi4, peptidyl arginine deiminase 4, involved in NETs formation and neutrophil function; pMphs, peritoneal macrophages; Poly(I:C), Polyinosinic:polycytidylic acid.

Of note, systemic routes of LM delivery do not mimic the natural route of its entry. Following a more physiologically relevant intragastric challenge, the absence of IFN–I signaling either had no beneficial effect (54) or even worsened LM infection (118). Furthermore, following systemic LM challenge, the deleterious effects of IFN–I were observed at relatively late stages of the infection; at early time–points (e.g., 24 hours post–infection), *Ifnar1^–/–^* and wild type (WT) mice showed no significant differences (117). Moreover, administration of IFN–β within the first 24 hours post–challenge enhanced protective responses, including increased IFN–γ production and natural killer (NK) cell cytotoxicity (125). A protective contribution of IFN–I has also been observed in MyD88^–/–^ mice, in which IFN–I signaling promoted the recruitment of protective Ly6C^hi^ monocytes to infection sites (124) (**Table 1**).

Overall, the role of IFN–I in LM infection is context–dependent: IFN–I contribute to protection or exacerbate the disease depending on the timing, the route of pathogen entry, and host immune background.

### 4.2 *Salmonella Typhimurium* infection

Salmonella generally causes acute infections, although it can also chronically persist. As with *LM*, a key element of host resistance is IFN–γ (126). Mice deficient in IFN–I signaling displayed higher resistance to *Salmonella enterica* serovar Typhimurium (ST) infection than WT mice (63, 65, 127, 128) (summarized in **Table 2**). A detrimental effect of IFN–I has been observed following systemic infection (127, 130) and, in some studies, also after the more physiologically relevant orogastric route of ST delivery (63, 65, 128). Administration of Poly(I:C) prior to ST infection exacerbated ST dissemination in an IFNAR1–dependent manner (63). The adverse effects of IFN–I have been attributed to the suppression of protective immune responses, including: (i) the induction of macrophage death via necroptosis or apoptosis (127, 129), (ii) the inhibition of IL–1β production (65), and (iii) the repression of neutrophil attracting chemokines (65). However, some studies have associated IFN–I–dependent susceptibility to ST with the exacerbation, rather than the inhibition, of the inflammatory responses. *Usp18^Ity9^* mice bear a loss–of function mutation of the *Usp18* gene, a negative regulator of IFN–I response. The mice exhibited increased susceptibility to ST infection due to elevated production of IFN–β, IFN–I–dependent overproduction of IL–10 and increased expression of a set of pro–inflammatory cytokines including IL–1β, IL–6 and IL–17 (130). Also, IFN–I can enhance *Salmonella* virulence by promoting lysosome acidification, which facilitates bacteria escape, survival and host cell death (63) (**Table 2**). Human studies support a pathological role for IFN–I: trancriptomic analysis of blood samples obtained from volunteers orally challenged with *Salmonella enterica* serovar Typhi revealed a strong IFN–I signature only in those participants who developed enteric fever, and this signature correlated with bacteremia and clinical markers of disease severity (133).

**Table 2:** The diversity of IFN–I–related effects during *Salmonella typhimurium* infection*.

Reference	Mice, background, cells	Challenge and other experimental details	Conclusions on IFN–I effects on:								
			Disease course	Th1 response, IFN–γ, T–cells	IL–1β	Proinflammatory cytokines/chemokines	IL–10	Mph/DC activation	Mph and DC recruitment	Neu recruitment	Immune cell viability
(127)	*Ifnar1^–/–^*, *Rip3^–/–^*, B6, BMDMs	ST: i.v., i.p., *in vitro*	D	NA	↓ IL–1β	NE: IL–6, IL–12	NA	NA	↓ CD11b^+^F4/80^+^ (spleen)	NE	RIP1/RIP3–dependent Mph death
(129)	*Ifnar1^–/–^*, *Atg7 ^–/–^*, B6, BMDMs	ST: *in vitro*	NA	NA	NA	NA	NA	NA	NA	NA	RIP3–mediated autophagy
(65)	*Ifnb^–/–^*, *MyD88^–/–^*, *Il10^–/–^*, B6, pMphs, THP–1	ST: oral, i.p. *In vitro*: ST + IFN–β	D	NA	↓ IL–1β, IL–18	↓ CXCL1, CXCL2, CXCL5, IL–8	↑ IL–10	NA	NA	↓ MPO (small bowel sections)	NA
(63)	Chimeric WT, *Ifnar1^–/–^*, B6, human IECs	ST: o.g., ST + Poly(I:C), CRISPR/Cas9 IEC screening	D	NA	NA	NA	NA	Change lysosome acidification	NA	NA	NA
(128)	*Stat1^–/–^, Stat2^–/–^*, B6	ST: i.g.	D	NE: serum IFN–γ	NA	NE: TNFα, IL–6, MCP–1, IL–12	NE: IL–10	NA	NA	NE: Neu numbers	NA
(130)	Usp18^ΔIty9^, DBA2, 129S1, B6	ST: i.v., *Mtb*: aerosol α–IFNAR1	D	NE	↑ IL–1β	↑ IL–6, IL–23, IL–17	↑ IL–10	NA	NA	NA	Deregulated autophagy
(70)	conditional FAK^–/–^, pMphs, BMDMs	ST: o.g., Poly (I:C), α–IFNAR1, *in vitro*	P	↑ IFN–γ by NK cells	NA	↑ CXCL9, CXCL10	NA	NA	NA	↑ Ly–6G^hi^ CD11b^hi^	NA
(67)	*cGAS^–/–^*, *Sting^–/–^, Ifnar1^–/–^*, *Tlr4^–/–^* B6, B10, pMphs, RAW264.7	ST: i.g., *in vitro* ST + TBK1 inhibitor	P	NA	NA	↑ IL–6, CXCL10	NA	NA	NA	NA	NA
(66)	Zebrafish larvae, RAW264.7	STM, STMΔspv: immersion, *in vitro*: RAW264.7	P	↑ IFN–γ	NA	↑ CXCL8	NA	NA	NA	↑ Neu	Autophagy
(131)	THP–1, MDDCs	*In vitro*: ST overexpressing cGAS	P	NA	NA	NA	NA	↑ DC–induced T–cytotoxicity	NA	NA	NA
(132)	*Tlr4^–/–^, Tlr3^–/–^*, *MyD88^–/–^*, Trif^LPS2^, B6	ST: o.g., YE: i.v., o.g. recIFNβ, Poly(I:C) α–IFNAR1	P	↑ IFN–γ by NK cells	NA	↑ IP–10	NA	NA	NA	↑ Gr–1^+^	NA

*For footnotes, color codes and specific labels, see footnotes to **Table 1**.

α–IFNAR1, anti–IFNAR antibodies; cGAS, cyclic gmp–amp synthase; DC, dendritic cells; IECs, intestinal epithelial cells; i.g., intragastric; MDDCs, monocyte–derived DCs; MPO, myeloperoxidase; *Mtb*, *Mycobacterium tuberculosis*; o.g., orogastric; PC, peritoneal cavity; pMphs, peritoneal macrophages; RIP1, receptor–interacting protein 1; RIP3, receptor–interacting protein 3; ST, *Salmonella Typhimurium*; STM, *S. Typhimurium* strain χ3306; Spv, Salmonella plasmid virulence (spv) locus; TRIF^LPS2^, mice with a mutation resulting in non–functional TRIF protein; Usp18^ΔIty9^ mice; mice bearing a loss–of–function mutation of Usp18; USP18, ubiquitin specific peptidase 18 a negative regulator of IFN–I signaling; YE, *Yersinia enterocolitica.*

Nevertheless, not all studies support a detrimental role of IFN–I during ST infection. Disruption of at least one component of cGAS–STING pathway worsened ST infection in mice, indicating a protective role for cGAS–STING signaling (67). In line with these data, ST overexpressing cGAS induced a stronger IFN–I response in human macrophages and DCs and enhanced T–cell cytotoxicity, a response associated with antimicrobial protection (131). *Salmonella’s* virulence factor *Spv* plays a key role in immune evasion; in human macrophages, the *spv* locus has been shown to inhibit IFN–I production. In zebrafish larvae, the ST strain bearing a deletion of the *spv* locus induced an elevated IFN–I response and a milder infection than WT ST (66).

One of the mechanisms that support ST intracellular survival involves the recruitment of host focal adhesion kinase (FAK) to SCV, FAK promotes ST survival by inhibiting autophagy. Conditional knockout of FAK in myeloid cells attenuated ST growth and, in parallel, it increased IFN–β production in macrophages. The administration of anti–IFNAR1 antibodies to ST–infected FAK^–/–^ mice significantly increased bacterial colonization, whereas pretreatment with poly(I:C) had a protective effect (70).

Sotolongo and co–authors analyzed the effect of IFN–I signaling on mouse susceptibility to Gram–negative enteropathogens, including ST, *Yersenia enterocolitica* and *E. coli*, using a *TRIF^–/–^* mouse model (132). The lack of TRIF–mediated signaling increased susceptibility to infections due to a deficiency in IFN–β production. *In vivo*, a single injection of poly(I:C) to *TRIF^–/–^* mice challenged with *Yersenia enterocolitica* reduced bacterial burden, whereas administration of anti–IFNAR1 antibodies abolished poly(I:C) beneficial effect. *In vitro*, macrophage–derived IFN–β stimulated NK cells to produce IFN–γ, which enhanced macrophage capacity to kill the bacteria (132).

In summary, IFN–I signaling generally reduces host resistance to ST and promotes pathology in immunocompetent host, but contributes to protection in the context of deficiencies in certain immune response–associated factors.

### 4.3 *Francisella* infection

Immune responses to *Francisella* have mainly been studied using low–virulence and vaccine strains. Both protective and pathological effects of IFN–I have been reported (**Table 3**). Evidence for a protective role of IFN–I is supported by two main observations. First, *in vitro*, recombinant IFN–β reduced bacterial growth in murine and human macrophages and induced IL–1β, TNF–α, IL–6 and KC (134) as well as T cell response–associated factors CXCL10, CCL5, IFN–γ, and iNOS (79) (**Table 3**). Second, IFN–I have been shown to activate the AIM2 inflammasome in macrophages (78, 80). AIM2 recognizes *Francisella* DNA and induces caspase–1–dependent processing of IL–1β, IL–18 and gasdermin–D, as well as pyroptotic cell death, all of which are known to contribute to protection (85). The protective role of AIM2 (and thus indirectly that of IFN–I) is supported by the reduced resistance of AIM2^–/–`^ mice to *F. novicida* infection (135, 137).

**Table 3:** The diversity of IFN–I–related effects during *Francisella* infections*.

Reference	Mice, background, cells	Challenge and other experimental details	Conclusions on IFN–I effects on:								
			Disease course	Th1 response, IFN–γ, T–cells	IL–1β	Proinflammatory cytokines/chemokines	IL–10	Mph/DC activation	Mph and DC recruitment	Neu recruitment	Immune cell viability
(79)	*Ifnar1^–/–^*, *Tlr2^–/–^*, B6, pMphs	*In vitro*: Ft LVS + IFN–β	P (*in vitro*)	↑ IFN–γ, mRNA	↑ IL–1β (protein)	NE: TNF-α, IL-12p35, IL12-p40, CXCL10, iNOS, CCL5	NA	NA	NA	NA	NA
(134)	*WT, MyD88–/–* B6, pMphs, RAW264, human MDMs	*In vitro*: Ft LVS + IFN–β	P (*in vitro*)	NA	↑ IL–1β	↑ IL–6, KC, TNF–α, CXCL2	NA	NA	NA	NA	NA
(135)	WT, *Ifnar1*^–/–^, *Ifnar2*^–/–^, *Aim2*^–/–^*Irf3*^–/–^*, Irf7*^–/–^*, Sting*^–gt/gt^, B6	*F. novicida*	D	NE: IFN–γ	↑ IL–1β, IL–18	NE: TNF–α, IL–6, IL–12p70, GM–CSF, CCL3	NE: IL–10				Caspase–dependent death
(136)	*Ifnar1^–/–^, Il17a*^–/–^, *WT, B6*	*F. novicida i.d.*, *Ft SCHU S4, i.n.*	D			↓ IL17A/F by γδ T cells				↓ CD11b^+^ Gr1^hi^ 7/4^hi^ F4/80^–^	

*For footnotes, color codes and specific labels, see footnotes to **Table 1**.

BMDMs, bone marrow derived macrophages; Ft LVS, *F. tularemia* live vaccine strain; Ft SCHU S4, *F. tularemia* SCHU S4 virulent strain; i.d., intradermal; i.n. intranasal; MDMs, human monocyte–derived macrophages; pMphs, peritoneal macrophages.

However, mice deficient in IFNAR, STING, or IRF3 exhibit increased resistance to *F. novicida* compared to WT mice (83, 135). Furthermore, *Ifnar*^–/–^*Aim2*^–/–^ mice also display increased resistance, indicating that the detrimental effects of IFN–I signaling outweigh the protective ones (135). A negative role of IFN–I in resistance to *Francisella* also follows from a higher level induction of *IFNB* expression by virulent *F. tularensis* SCHU4 strain compared with the attenuated *F. novicida* strain in human monocytes (82). Analyses of immunological mechanisms underlying IFN–I deleterious effects have identified neutrophils as the main target population. IFN–I were shown to suppress neutrophil inflammation by inhibiting IL–17 production by γδ T cells (136) and by inducing cell apoptosis through apoptotic caspases (135). It should be noted, however, that although neutrophils are essential for protection against *Francisella* (138, 139), excessive neutrophilic recruitment can exacerbate pulmonary tularemia (140).

Clinical data on IFN–I during natural human tularemia are limited. Most human studies have analyzed immune responses to *F. tularensis* LVS and did not specifically evaluate IFN–I. The most informative dataset comes from the study by Andersson and co–authors (141) who performed whole–blood transcriptional profiling of patients with acute ulceroglandular tularemia. The dominant signature was, however, associated with IFN–γ leaving a role of IFN–I during tularemia uncertain. Overall, available data on IFN–I during *Francisella* infections are largely based on animal or *ex vivo* models, and responses to low–virulence strains.

### 4.4 *Mycobacterial* infections

In contrast to LM and ST infections, mycobacterial infections are typically chronic. Early protection depends on the successful production of pro–inflammatory cytokines, effective recruitment of innate immune cells to the infection site, and generation of *Mtb*–specific Th1 responses (142–145). If mycobacteria are not cleared, the infection may persist in a latent form or progress to active disease. The outcome largely depends on the host’s capacity to fine–tune inflammatory responses, as the same cells and mediators that provide protection can become deleterious if uncontrolled (146–150). IFN–I possess both pro– and anti–inflammatory activities and could theoretically fulfil an immunoregulatory function during TB. However, research findings indicate that IFN–I predominantly exert pathological effects (summarized in **Table 4**).

**Table 4:** The diversity of IFN–I–related effects during mycobacterial infections*.

Reference	Mice, background, cells	Challenge and other experimental details	Conclusions on IFN–I effects on:								
			Disease course	Th1 response, IFN–γ, T–cells	IL–1β	Proinflammatory cytokines/chemokines	IL–10	Mph/DC activation	Mph and DC recruitment	Neu recruitment	Immune cell viability
(87)	B6D2/F1, *Ifnar1^–/–^*, A129	*Mtb* clinical isolates, aerosol, α–IFNαβ	D	NA	NA	↓ IL–12	NA	NA	NA	NA	NA
(151)	*Ifnar1^–/–^*, B6	*Mtb* aerosol, *Mtb* + LCMV, Poly(I:C), α–IFNAR1, scRNA–seq	D	↓ IFNγ^+,^TNFα^+^ IFNγ^+^ CD4^+^ & CD8^+^	NA	↑ TNF–α, IL–6, CCL2, CXCL1, CXCL5 (lungs)	NE	↓ *Cxcl9, Cxcl10* in Mphs	NA	↑ CD11b^+^Ly–6G^+^ (lungs)	NA
(88)	B6	*M. bovis*, i.n., α–IFNAR1 (day–1)*	D	↓ IFN–γ (lungs)	↓ IL–1β	NE: TNF–α, IL–12, IL–4, IL–17, ↑ IL–6	↑ IL–10	↓Nos2, ↑ Ym1, Arg1, CD206	NA	↑ Ly6G^+^Ly6C^+^	NA
(152)	*Ifnar1^–/–^*, *Ccr2^–/–^*, B6	*Mtb*, aerosol + Poly–ICLC i.n., prolonged	D	NE: T–bet, IFNγ^+^TNFα^+^, CD4^+^CD44^+^	NA	NA	NA	NA	↑ permissive CD11b^+^Gr1^int^		↓ (induce lung tissue necrosis)
									NE: CD11b^+^Gr1^–^	NE: CD11b^+^Gr1^hi^	
(153)	*Ifnar1^–/–^* B6 *Ifnar1^–/–^* 129S2	*Mtb*, aerosol	D	NE	↑ IL–1β	↑ TNF–α, IL–6, CXCL1, CXCL5, CCL2, CCL4, G–CSF	NA	NA	↑ CD11b^+^ Ly6C^+^	↑ CD11b^+^Ly6G^+^	↓ in Mphs ↑ in Neu
(154, 155)	B6, B6.sst1^s^, *Ifnar1^–/–^*, *SP140^–/–^, SP140^–/–^Ifnar1^–/^*^–^	*Mtb*, aerosol	D	NE: IFN–γ	↓ IL–1β (↑ *Il1rn*)	↑ TNF–α, CXCL1	NE	NA	NA	↑ CD11b^+^ Ly–6G^+^	NA
(156)	*Ifnar^–/–^*, *Tpl2^–/–^*, *Rag1^–/–^*, *Tpl2^–/–^Ifnar^–/–^*, B6, BMDMs	*Mtb*; aerosol; LM i.v. *in vitro*	D	NA	NA	↓ IL–12	↑ IL–10	↓ *Ifngr*	NA	NA	NA
(157)	*Ifnar^–/–^*, *Nos2^–/–^*, *Il10^–/–^*, *Tcrr^–/–^*, B6, BMDMs, lung Mphs	*In vitro*; recIFN–β i.n.	D	NA	↓ IL–1β	TNFα, IL–12: ↓ by recIFNβ, ↓in Ifnar^–/–^	↑ IL–10	↓ response to IFN–γ, ↓ killing	NA	NA	NA
(158)	*Gsdmd^–/–^*, *Stat2^–/–^*, *Casp1^–/–^*, *Casp11^–/–^*, *Casp1^–/–^Casp11^–/–^*, B6, 129S2, RAW264.7	*Mtb*, aerosol, Rif + anti–IFNAR, CRISPR/Cas9 GWS	D	NA	NA	NA	NA	NA	NA	NA	↓ (induce Mphs death)
(159)	*Stat1^–/–^*, *Ifnar1^–/–^*, *Ifngr^–/–^*, *Ifngr^–/–^Ifnar^–/–^*, B6	*Mtb*, aerosol	P in *Ifngr ^–/–^*	NE: NK, CD4^+^, CD8^+^	NA	NA	NA	NA	↓ infected CD11b^+^Gr1^low^	↓ infected CD11b^+^Gr1^hi^	NA
(160)	*Ifnar1^–/–^*, *Il1r1^–/–^*, *Ifnar1^–/–^ Il1r1^–/–^*, *Nos2^–/–^*, B6,	*rpoB*–H445Y *Mtb*	P in *Il1r*^–/–^	NA	NA	NA	NA	↑ NO	NA	NA	NA
(161)	*Ifnar1^–/–^*, *Nos2^–/–^*, *Ifngr^–/–^*, B6, BMDMs, lung Mphs	*In vitro: Mtb*, *Msm, M. bovis*	P	NA	NA	NA	NA	↑ NO, *Mtb* killing	NA	NA	NA
(162)	Zebrafish larvae: WT, *Stat2^–/–^*, TST skin biopsy	*M. marinum*, TST transcriptome	P	NA	NA	NA	NA	NA	NA	↑ Neu	NA

For footnotes, color codes and specific labels, see footnotes to **Table 1**. α–IFNαβ, anti–IFN–α/IFN–β antibodies; α–IFNAR1, antibodies against the IFN–α/β receptor; *Arg1*, Arginase 1; B6, C57Bl/6 mice; B6.sst1^s^, mice harboring the susceptible haplotype of *Sst1* from the C3H/HeBFeJ strain; BCG, *Bacillus Calmette–Guérin*; BMDMs, bone marrow–derived macrophages; CRISPR/Cas9 GWS, genome–wide screening using CRISPR/Cas9; DC, dendritic cell; i.n., intranasal; LCMV, lymphocytic choriomeningitis virus; *M. bovis, Mycobacterium bovis, M. marinum, Mycobacterium marinum*; MDMs, monocyte–derived macrophages; Mphs, macrophages; *Msm, Mycobacterium smegmatis; Mtb, M. tuberculosis*; Neu, neutrophils; NK, natural killer cells; NO, nitric oxide; Nos, nitric oxide synthase; recIFN–β, recombinant IFN–β; Rif, rifampicin; *rpoB*–H445Y *Mtb, Mtb* carrying an rpoB H445Y SNP conferring drug resistance; scRNA–seq, single–cell RNA sequencing; SP140, speckled protein 140, a negative regulator of IFN–I during bacterial infections; Sst1, s*uper susceptibility to tuberculosis 1* locus; T–bet, T–box transcription factor; Tccr^−/−^, IL–27R α–chain–deficient mice; Tpl2, tumor progression locus 2, a MAP3K family member; TST, tuberculin skin test.

In mice, the more virulent clinical isolate of *Mtb*, NH878, elicited a stronger IFN–I response than less virulent isolates (163). Prolonged intranasal (i.n.) administration of purified IFN–α/β, Poly(I:C) or Poly(I:C) condensed with poly–L–lysine and carboxymethylcellulose (Poly–ICLC) exacerbated experimental *Mtb* infection (151, 152, 157, 163). Deletion of *Ifnar1* or the administration of anti–IFNAR antibodies, either as monotherapy (88) or in combination with Rifampicin (158) were beneficial. Immunological mechanisms proposed to underlie the deleterious effects of IFN–I include: (i) inhibition of T–cell proliferation and suppression of the development of antigen–specific Th1 and CD8 T cells (88, 151); (ii) inhibition of IL–1β (88, 154, 155, 157); (iii) inhibition or stimulation of TNF–α, IL–6 and IL–12 (with various results obtained in different studies) (151, 153, 154, 156, 157); (iv) stimulation of IL–10 (3, 88, 156, 157); (v) macrophage polarization towards an anti–inflammatory profile (88); and (vi) modulation of the recruitment of permissive or protective myeloid cells to the site of infection (observed in most studies) (88, 151–155, 162) (summarized in **Table 4**). Similar effects were registered in *in vitro* models of macrophage infection (90, 157).

Viral infections induce a robust IFN–I response. Pre–challenge of mice with influenza A virus or infection with lymphocytic choriomeningitis virus (LCMV) during ongoing *Mtb* infection worsened *Mtb*–induced disease due to the enhanced IFN–I response (151, 164). Immunological consequences of LCMV co–infection included: (i) inhibition of CXCL9 and CXCL10 production by macrophages and the subsequent reduction in the recruitment of *Mtb*–specific IFN–γ–producing CD4 and CD8 T cells; (ii) an increase in the levels of inflammatory cytokines and chemokines IL–6, TNF–α, CCL2, CXCL1, and CXCL5 in lung homogenates; and (iii) enhanced accumulation of CD11b^+^Ly–6G^+^ neutrophils in the lungs (151). Tumor progression locus 2 (*Tpl2*) negatively regulates the production of multiple cytokines, including IFN–I. In the absence of *Tpl2*, excessive levels of IFN–I exacerbated experimental TB infection via IFN–I–dependent promotion of IL–10 production (156). Noteworthy, the effects were seen at the chronic (days 56–100), but not at the early (day 28) stage of infection.

Mice of different genetic backgrounds differ by their susceptibility to *Mtb* (165, 166). In 129S2 mice, deletion of *Ifnar1* enhanced resistance to *Mtb*, due to a reduced activation of NF–κB, lower production of pro–inflammatory cytokines IL–1β, IL–1α, TNF–α, IL–6, G–CSF, CXCL1, CXCL5, CCL2, CCL4 and CCL5 and a reduced accumulation of neutrophils (CD11b^+^Ly6G^+^ cells) and inflammatory macrophages (CD11b^+^Ly6C^+^ cells) in the lungs (153). B6.sst1^s^ mice carry the susceptible allele of the S*uper susceptibility to tuberculosis 1 (Sst1*) locus that confers susceptibility to *Mtb* by enhancing IFN–I response (167). Similar to 129S2 mice, deletion of *Ifnar1* in B6.sst1^s^ mice was also protective, but this protection was achieved through mechanisms opposite to those observed in 129S2 mice: it reduced the expression of IL–1 receptor antagonist *Il1rn* and increased IL–1β activity, a prerequisite for TB protection (154). The *Sst1* locus contains the *SP140* gene, which represses IFN–I transcription. *SP140^–/–^* mice showed elevated expression of *Ifnb*, leading to increased susceptibility to *Mtb* and *Legionella pneumophila* infections associated with elevated expression of *IL–1rn* (155).

Unlike in other bacterial intracellular infections, in mycobacterial infections, only a few studies have observed a beneficial effect of IFN–I. Desvignes and co–authors have found that IFN–I signaling is protective in the absence of the IFN–γ signaling (159). Khadar’s group observed a protective effect of the IFN–I in mice and mouse bone marrow–derived macrophages (BMDMs) lacking IL–1 signaling and infected with rifampin–resistant *rpoB*–H445Y *Mtb* (160). This effect was attributed to increased nitric oxide production, a phenomenon also observed by other authors (161).

Clinical observations largely (although not uniformly) support a detrimental role for IFN–I during mycobacterial infections. The clinical manifestations of leprosy range from self–healing to disseminated disease form. In progressive lepromatous lesions, the expression of IFN–β is higher than in self–healing tuberculoid lesions (168). Patients with active TB were shown to differ from latently infected individuals by higher–level expression of IFN–I–inducible genes (86, 169–173). Moreover, in *Mtb*–infected individuals, IFN–I was upregulated as early as 18 months prior to TB diagnosis, indicating a causal role for IFN–I in TB progression (172, 174). However, associations do not necessarily imply cause–and–effect relationships, i.e., IFN–I signature detected in TB patients, even prior to TB diagnosis, can be a consequence rather than a cause of TB activation. Furthermore, recently, Szydlo–Shein and colleagues have found that reduced IFN–I signature in tuberculin–skin–test (TST) transcriptome is associated with increased severity of human TB disease (162). In the study by Llibre and co–authors (175), TB patients differed from asymptomatic *Mtb*–infected individuals in that they had elevated levels of ISGs. However, no increase in IFN–α/IFN–β levels was observed in their plasma. Given that ISGs may be induced by several non–canonical mechanisms (176), the data raise a question as to whether the induction of IFN–I signature during active TB is a direct consequence of IFN–I over–production.

A more direct indication on the involvement of IFN–I in human TB activation and pathology comes from case reports showing an increased risk of TB development in patients receiving IFN–α for hepatitis C treatment (177, 178) or IFN–β as a part of multiple sclerosis treatment (179). However, when considering the latter data, it should be borne in mind that multiple sclerosis treatment usually includes not only IFN–β, but also other immunosuppressive drugs. Furthermore, early studies reported a beneficial therapeutic effect of IFN–α if it was used in combination with antimycobacterial therapy (180, 181).

Altogether, a general consensus is that during mycobacterial infections, IFN–I are pro–pathogenic. One recent paper even suggested considering TB as an unconventional interferonopathy (182). Yet, even in the context of *Mtb* infection, some studies have reported IFN–I protective effect (159, 160, 162). Data on immunological mechanisms underlying IFN–I effects are highly controversial. The exception is perhaps IFN–dependent stimulation of myeloid cell recruitment, for which different groups have obtained largely similar results (**Table 4**).

### 4.5 Extracellular bacterial infections

Data on the impact of IFN–I in resistance to PA are inconsistent (**Table 5**). In some studies, reduced IFN–α/β production aggravated pulmonary pathology, accelerated mortality, and increased the production of pro–inflammatory cytokines, including IL–1β and TNF–α (105, 190). In other studies, however, intact IFN–I signaling was detrimental, due to IFN–I–dependent neutrophil activation, elevated ROS production, and enhanced NET formation, which promoted PA biofilm development (187, 191).

**Table 5:** The diversity of IFN–I–related effects during infections elicited by extracellular bacteria*.

Reference	Mice, background, cells	Challenge and other experimental details	Conclusions on IFN–I effects on:									
			Disease course	Th1 response, IFN–γ, T–cells	IL–1β	Proinflammatory cytokines/chemokines		IL–10	Mph/DC activation	Mph and DC recruitment	Neu recruitment	Immune cell viability
(39)	*Ifnar1^–/–^*, *Irf1^–/–^, Irf3^–/–^*, *Irf5^–/–^, Tlr3^–/–^, Tlr7^–/–^, Tlr9^–/–^, MyD88^–/–^, MAVS^–/–^*, *Trif^–/–^*, B6, BMDMs, cDCs	*S. pyogenes*, s.c.	P	NA	NA	NA	NA		NA	NA	↓ Neu	NA
(183)	*Ifnar1^–/–^*, *Ifnar1^fl/fl^ Lysm–Cre*, *Ifnar1^fl/fl^ Cd11c–Cre*, B6	*S. pyogenes*, s.c.	P	NA	↓ IL–1β	NE: TNF–α, IL–12, CXCL1, IL–1α	NE: IL–10		NA	NA	↓ Ly–6C^+^Ly6G^+^	NA
(97)	*Ifnar1^–/–^Ifngr^–/–^*, *Ifnar1^–/–^*, *Ifngr^–/–^, Ifnb^–/–^*, 129Sv, B6	GBS i.c. or i.p. SPn, *E.coli* i.v.	P	↑ IFN–γ by Mphs		↑ TNF–α by Mphs	NA		↑ NO by Mphs	NA	NA	NA
(99)	*Tlr4^–/–^*, *Nod2^–/–^, MyD88^–/–^*, *Trif^–/–^, Ifnar1^–/–^*, B6, 129Sv/Ev	SPn i.n., *In vitro*	P	NA	NA	NA	NA		NA	NA	NA	NA
(184)	*Ifnar1^fl/fl^ Lysm–Cre*, *Ifnar1^fl/fl^Cd11c–Cre, Ifnar1^fl/fl^ Sftpc–Cre*, B6	SPn i.n. 16–40h	P	NA	↓ IL–1β (lung)	↓ CXCL1, TNF–α (lung, plasma)	NA		NA	↓ Mon/Mphs	NA	↑AECII viability
(185)	B6	SPn i.t. Ad5–IFNα	P	NA	NA	20h p.i.:↑ IL–1β, IP–10, MIP–2, MCP–1	NA		*In vitro*: ↑ SPn killing	20h p.i.: NE CD11c^+^Gr–1^+^	20h p.i.: ↑ CD11b^+^Gr–1^+^	NA
						72h p.i.: ↓ TNF–α, IP–10, MIP–2, KC, MCP–1				72h p.i.: ↓ CD11c^+^Gr-1^+^	72h p.i.: ↓ CD11b^+^Gr-1^+^	
(186)	*Ifnar1^–/–^*, *Ifnar1^fl/fl^Lysm–Cre*, *Ifnar1^fl/f^ Cd11c–Cre*, *Ifnar1^fl/fl^Mrf8–Cre*, B6	*KP i.n.*	P	↑ CD4+, CD8+, NK1.1^+^ IFN–γ^+^	NA	↑ IL–12, CXCL10, TNF–α (lungs)	NE: IL–10		NA	NE	NE	NA
(105)	*cGAS^–/–^, STING^–/–^*, B6 RAW264.7, THP–1, MH–S	PA i.n.	P	NA	↓ IL–1β	↓ TNF–α, IL–6	NA		NA	NA	NA	NA
(187)	*Ifnar1^–/–^, Ifnb^–/–^*, B6	PA i.t.	D	NA	NA	↑ TNF–α	NA		NA	NA	↑ NETosis ↑ PA biofilms	NA
(114)	*Ifnar^–/–^*, 129Sv/Ev, B6, mAECs, hAECs	SA i.n.	D	NA	NA	NE: IL–6, modulate TNF–α	NA		NA	↓ CD11c^+^ DCs	NE: Neu	NA
(110)	*Ifnar1^–/–^*, *Tnfr^–/–^, Irf1^–/–^, Tlr7^–/–^*, *Tlr9^–/–^, MyD88^–/–^*, B6, BMDCs	SA (MRSA) i.n., *In vitro*	D	↓CD4^+^	NA	↑ TNF–α, NE: IL–6, CXCL10, IL-17	NA		NA	NE: Mphs, DCs	NE: Neu	NA
(188)	ISG^hi^,& ISG^low^ neutrophils from ARDS patients	SA *in vitro*	D	NA	NA	↓ IL–8 in Neu	NA		NA	NA	↓ migration & SA killing	Neu: ↓ necrosis, ↑ apoptosis
(189)	*Ifnar1^–/–^*, B6, BMDMs	SA i.n.	D	NA	NA	NA	↓ IL–10		NA	NA	NA	↑ Neu/Mph apoptosis

*For footnotes, color codes and specific labels, see footnotes to **Table 1**.

Ad5–IFN–α, Ad5 expressing IFN–α; AECII, type II alveolar epithelial cells; ARDS, acute respiratory distress syndrome; BMDCs, bond–marrow–derived dendritic cells; cDCs, conventional dendritic cells; GBS, group B streptococcus; hAECs, human alveolar epithelial cells; i.n., intranasal; i.p., intraperitoneal; i.t., intratracheal; i.v., intravenous; *KP, Klebsiella pneumonia*; mAECs, mouse alveolar epithelial cells; Mon, monocytes; Mphs, macrophages; NETosis, formation of neutrophil extracellular traps; Neu, neutrophils; s.c., subcutaneous; SA, *S. aureus*; SPn, *S, pneumonia*.

In KP infection, the absence of IFN–I signaling reduced mouse survival and increased lung bacterial burden, primarily due to impaired IL–12 and CXCL10 production by macrophages, decreased IFN–γ production by NK cells (186), and diminished cytotoxic and Th1 functions of mucosal–associated invariant T cells (MAIT) (192).

In SPy infection, IFN–β produced by LysM^+^ and CD11c^+^ myeloid cells contributed to protection by limiting inflammation. *Ifnar1^–/–^* mice challenged with *SPy* had shorter survival times, elevated IL–1β and inflammatory markers (such as alanine transaminase, aspartate aminotransferase, and creatinine) and increased neutrophil recruitment. The expression of *Tnfa*, *Cxcl1* and *Il10* was independent of IFN–I signaling in this model (39, 183).

In SPn infection, IFN–I–mediated protection occurred via suppression of IL–1β, TNF–α and CXCL1, and the consequent protection of alveolar epithelial type II cells (AECII) from inflammation–induced death (184). Damjanovic and co–authors suggested that IFN–I act as immune regulators rather than purely anti–inflammatory mediators: adenovirus 5 expressing IFN–α administered prior to SPn challenge increased IL–1β, IP–10, MCP–1, and MIP–2 production and neutrophil recruitment to the lungs at 20h post–infection, but reduced or terminated these responses by 72 h post–infection, likely due to IFN–α–dependent reduction in bacterial burdens (185). The pro–inflammatory action of IFN–I on myeloid cells was also demonstrated *in vitro*: *Ifnar1^–/–^* and *Ifnb^–/–^* macrophages produced significantly less TNF–α, IFN–γ and nitric oxide in response to group B *Streptococcus* compared with WT macrophages (97).

Overall, unlike in intracellular bacterial infections, IFN–I are generally protective in most infections caused by extracellular bacteria (**Table 5**).

### 4.6 Infection with *Staphylococcus aureus*

Differentially from many typical extracellular infections, IFN–I effects during SA infections are context–dependent (**Table 5**). In a subcutaneous SA infection model, IFN–β promoted infection control, enhancing SA clearance and reducing lesion sizes (193). In a MRSA skin infection model, inhibition of SOCS1 increased bacterial phagocytosis and killing and decreased lesion size and bacterial loads. These beneficial effects were IFNAR–dependent and abrogated by IFNAR deletion or antibody blockage (194). In contrast, during respiratory SA infection, IFN–I appear detrimental. The absence of IFN–I signaling improved SA clearance by reducing TNF–α production (110) and enhancing the recruitment of CD11b^+^CD11c^+^ DCs (114). *In vitro*, IFN–I stimulated IL–10 production and expression of pro–apoptotic genes by mouse BMDMs (189). Secondary post–influenza bacterial pneumonia caused by SA is a severe complication of primary influenza virus infection (195). Enhanced susceptibility to SA and MRSA superinfection has been linked to influenza–induced IFN–I overproduction, which suppresses KC, MIP–2 and IL–1β production, reduces Th17 responses, diminishes neutrophil recruitment, and promotes M2–biased macrophage differentiation (196–200). In patients with acute respiratory distress syndrome (ARDS), neutrophils displayed heterogeneity in ISG expression. Neutrophils with high ISG levels were functionally impaired, producing less IL–8, showing reduced migratory and bactericidal capacities, and being more prone to cell death (188). Thus, tissue localization influences whether IFN–I are protective or pathological during SA infection. Mechanisms underlying these differences likely include inter–tissue variations in immune populations, IFN–I levels, the nature and magnitude of other inflammatory responses and additional factors that are yet to be determined.

## 5 Immunological consequences of IFN–I signaling during bacterial infections

As the studies reviewed above demonstrate, IFN–I exert a wide range of effects across bacterial infections. In this section, we summarize these effects based on their immunological consequences rather than by pathogen. The effects that have already been thoroughly reviewed (3, 11, 201, 202) are addressed only briefly here, while more detailed consideration is given to the impact of IFN–I on myeloid cells (see **Box 2** for a concise summary of IFN–I effects).

Box 2 **Main immunological and hematopoietic effects of IFN–I.**

Proinflammatory and immunoregulatory cytokines
IFN–I promote the production of IL–10. Depending on the context, IFN–I can inhibit or stimulate the production of IL–12 and TNF–α. On IL–1β, IFN–I mainly exert an inhibitory effect.
IFN–γ and Th1 responses
IFN–I potentiate IFN–γ production by innate immune cells, particularly, NK cells, at early infection stages and can inhibit IFN–γ synthesis and Th1 development under conditions of high IFN–I doses or chronic IFN–I exposure.
Macrophages
IFN–I inhibit macrophage expression of IFNGR and reduce cell responsiveness to IFN–γ attenuating macrophage antimicrobial activity. They also promote various mechanisms of macrophage death, including apoptosis, necrosis, and other, less well–characterized mechanisms.
Dendritic cells
IFN–I accelerate DC differentiation from monocytes in vitro and promote maturation of immature DCs, increasing their expression of MHCII and costimulatory receptors, cross–presentation and Th1–inducing capacities. On mature DCs, IFN–I mainly exert an immunosuppressive, anti–inflammatory effect.
Neutrophils
IFN–I can both support neutrophil survival and promote their death, they also stimulate the formation of NETs, which may promote bacterial growth rather that suppress it.
Myeloid cell recruitment
IFN–I can both stimulate and inhibit myeloid cell recruitment to, and the accumulation at, the site of infection. Stimulation of monocyte/macrophage recruitment is mediated by upregulation of CCR2 and increased secretion of CCL2. Inhibitory effects are largely due to enhanced macrophage death. Modulation of neutrophil recruitment largely depends on the regulation of CXCL1, CXCL2, and IL17 expressions.
Hematopoietic stem and progenitor cells (HSPCs)
Under physiological conditions, IFN–I maintain HSPC pool. Under high IFN–I doses, HSPCs lose quiescence and start cycling, which impairs their self–renewal and promotes differentiation. In adults, differentiation is switched toward myeloid lineage, can occur via a shunted pathway, and can preferentially generate mononuclear or granulocytic cells depending on the context.
Trained immunity
Emerging evidence indicates that IFN–I can directly induce trained immunity, as well as participate in the formation of trained immunity induced by other stimuli.

### 5.1 Cell–mediated antibacterial defense mechanisms

The antibacterial immune response develops as a complex, multistage process. In the initial stage, innate immune cells, primarily of myeloid origin, are activated and begin producing pro–inflammatory cytokines and chemokines, which amplify innate immune reactions and orchestrate the development of adaptive immune responses. In the lymph nodes, antigen–specific T–lymphocytes interact with antigen–presenting cells, undergo activation, expansion and differentiation into polarized populations of effector antigen–specific T–cells, primarily Th1, Th17 and CD8 effectors. These cells subsequently migrate to the site of infection, where they accomplish their effector functions directly and/or by activating innate immune cells. In parallel, and depending on the type of pathogen, antibody–producing plasma cells are generated and provide antibody–dependent bacterial neutralization and clearance. The generation of effector responses is accompanied by the formation of T– and B–cell memory (203). If the pathogen is cleared, the inflammation will resolve, otherwise, it progresses to a chronic stage. Both resolution and chronicity of inflammatory reactions are immunologically active processes (204, 205). The overall efficacy of antibacterial defense depends on the effectiveness of each stage, including the host’s capacity to prevent excessive inflammatory reactions. IFN–I can modulate all of these processes.

### 5.2 Pro–inflammatory and immunoregulatory cytokines

#### 5.2.1 IL–12 and TNF–α

IL–12, TNF–α and IL–1β are key cytokines for antibacterial protection. IL–12 is produced by antigen–presenting cells and is essential for the generation of Th1 lymphocytes (206). The general consensus is that IFN–I inhibit IL–12 production (117, 207–211). This effect is dependent on STAT1/STAT2 and PI3K signaling (211). However, an IL–12–stimulatory effect of IFN–I; particularly in immature DCs, has also been observed (3, 186, 212–214).

TNF–α mediates protection by stimulating the production of other cytokines, recruiting immune cells to the sites of infection, and, in some cases, by promoting bacteria killing; it can also act as a negative regulator of Th1 responses, and it contributes to tissue pathology and disease exacerbation (215–219). A number of studies have documented suppressive effects of IFN–I on TNF–α (3, 62, 105, 184, 210, 220). However, in some settings, IFN–I exerted stimulatory and cross priming activities (i.e., an exposure to a low–dose IFN–I enhanced cellular response to TNF–α) (97, 151, 186, 221–223).

#### 5.2.2 IL–1β

Experiments in *Il1a^–/–^*, *Il1b^–/–^* and *Il1r1^–/–^* mice have shown that IL–1α and IL–1β are essential for efficient bacterial control (224–227). Both cytokines trigger the same receptor (227). The mechanisms underlying IL–1α/IL–1β effects are diverse and have been most extensively studied for IL–1β. In T lymphocytes, IL–1β promotes type 1 response (228) and stimulates secretion of IL–26, a human protein with direct antimicrobial pore–forming activity (229). In combination with IL–2, IL–1β drives the expansion of T–lymphocytes and NK cells (230). IL–1β also amplifies B–cell responses (231, 232) and triggers COX–2 activation and prostaglandin E2 synthesis, factors associated with a milder course of intracellular bacterial infections (233). In macrophages, IL–1β promotes M1 polarization, induces efferocytosis, supports bacterial containment in phagolysosomes and prevents pathogen transition to the cytosol (234, 235). Additional myeloid effects of IL–1β include the induction of emergency granulopoiesis (236), promotion of the differentiation of conventional DCs (232) and stimulation of myeloid cell recruitment to the infectious sites (237).

IFN–I suppress the production of IL–1α/β, which is often considered a key mechanism of IFN–I detrimental action (65, 88, 105, 154, 183, 184). Suggested mechanisms include: (i) IFN–I–dependent induction of IL–10, (ii) suppression of NLRP3 and NLRP1 inflammasomes and the inhibition of IL–1β cleavage via a STAT1–dependent mechanism (238, 239), (iii) induction of IL–1Ra, the IL–1 receptor antagonist (154, 240) and (iv) limitation of the *IL1B* expression at the mRNA level (90). Despite generally consistent evidence for IFN–I–mediated suppression of IL–1β, IFN–I–dependent increase in IL–1β production has also been reported (130, 153).

#### 5.2.3 IL–10

IL–10 is an immunoregulatory cytokine that antagonizes IL–12 in the induction of Th1 responses (241). The capacity of IFN–I to stimulate IL–10 production has been observed both *in vitro* and *in vivo* and is often considered as a mechanism responsible for IFN–I–mediated inhibition of host protection (54, 88, 157, 207, 211, 242, 243). As illustrated in **Tables 1**-**4**, studies that reported a stimulatory effect of IFN–I on IL–10 also observed a detrimental impact of IFN–I on the course of infection. However, not all studies that evaluated IL–10 levels in the context of bacterial infections detected IFN–I–dependent modulation of IL–10 production (128, 151, 154, 183, 186).

### 5.3 IFN–γ and Th1 response

IFN–γ produced by NK cells, Th1 and CD8^+^ T lymphocytes is central to controlling various infections, especially those caused by intracellular bacteria (244).

IFN–I have a complex, context–dependent role in regulating IFN–γ responses, combining both inhibitory and stimulatory effects. Acting on innate immune cells at an early infection stage, IFN–I stimulate IFN–γ production by adjacent NK and γσ–T–cells. The effect has been observed in both *in vivo* and *in vitro* models of ST, LM, *Y. enterocolitica*, *Chlamydia pneumoniae* and KP infections (66, 70, 125, 132, 186, 245). Some studies also report a stimulatory effect of IFN–I on IFN–γ–producing Th1 cells and cytotoxic CD8^+^ lymphocytes (246). The early stimulatory activity of IFN–I has been attributed to accelerated maturation of DCs (discussed below) and the formation of STAT4 homodimers, which induce T–bet, the key Th1–associated transcription factor (207, 247, 248). However, STAT4 activation by IFN–I is transient (249), making IFN–I–dependent stimulation of IFN–γ also transient, relatively weak, and mostly associated with acute infections.

Chronic and/or severe infections induce sustained high–level production of IFN–I, which favors inhibition of Th1 responses. Suppression of IFN–γ^+^ and polyfunctional IFN–γ^+^TNF–α^+^ CD4^+^ T cells and memory CD8^+^ T cells has been observed during *Mtb*, LM and *Francisella* infections, and has been attributed to: (i) suppression of IL–12, (ii) induction of IL–10, and (iii) stimulation of checkpoint inhibitors (3, 62, 88, 121, 151, 208, 250–253).

Although the data suggest that the effects of IFN–I on IFN–γ/Th1 are stage–dependent, many other factors can contribute to the net effect of IFN–I. These include pathogen biology, pathogen tissue and intracellular localization, the type of infection (acute versus chronic), the target immune cells (NK, DCs, macrophages, T cells), the levels of other cytokines (IL–1β, IL–10, IL–12, etc.) in cellular mileu, and the levels of IFN–I themselves. The latter factor has been insufficiently studied, but may play a key role in determining the outcomes of IFN–I responses. Notably, not all studies reporting a detrimental effect of IFN–I in bacterial infections observed repression of Th1 responses, indicating that such repression is not the sole mechanism underlying IFN–I action (**Tables 1**-**5**) (59, 128, 152–154).

As with Th1 responses, both inhibitory and stimulatory effects of IFN–I on Th2 and Th17 responses have been documented, further highlighting the ambiguous nature of T–cell–modulatory activities of IFN–I and their dependence on the infectious context (254–260).

### 5.4 Mononuclear myeloid cells

Mononuclear myeloid cells are key components of antibacterial protection. Their antibacterial activities are stimulated by IFN–γ and modulated by IFN–I (261–263).

#### 5.4.1 Repression of macrophage activation

In their pilot study back from 1980, Lee and Epstein reported that leukocyte interferon delays the maturation of monocytes to macrophages *in vitro*: IFN made the cells smaller in size, less stretched out and reduced their lysosomal enzymes’ activity (264). Further studies showed that IFN–I repress macrophage activation by IFN–γ (122, 156, 157, 210). The effect was due to the inhibition of the expression of IFN–γ receptor (IFNGR1) (122). The role of IFNGR1 in IFN–I–mediated repression of macrophage activity was directly documented by Eshleman and co–authors who generated transgenic mice expressing a functional FLAG–tagged *IFNGR1* (fGR1) under a macrophage–specific promoter. In fGR1 macrophages, IFN–I failed to inhibit cell responsiveness to IFN–γ, and these macrophages exhibited a significantly increased resistance to LM (265). Recently, single–cell RNA sequencing has been applied to identify lung cells expressing IFN–I and those responding to IFN–I during experimental *Mtb* infection. Plasmacytoid pDCs appeared as the main IFN–I producers, whereas interstitial macrophages were the main IFN–I–responsive cells; their exposure to IFN–I inhibited cell reactivity to IFN–γ and restricted *Mtb* control (266). Reciprocal relationships between IFN–I and IFN–γ have also been revealed in clinical settings, in the context of leprosy infection, where IFN–γ gene expression program prevailed in self–healing lesions, whereas IFN–β signature predominated in progressive lepromatous lesions (168).

Mechanisms suggested to underlie IFN–I–dependent inhibition of macrophage reactivity to IFN–γ include: (i) the formation of IFN–I–dependent STAT1/STAT2 heterodimers, which reduce the formation of STAT1 homodimers implicated in IFN–γ signaling, (ii) the induction of the protein inhibitor of activated STAT1 (PIAS1), and (iii) the activation of methyltransferases, which reduce the expression of several genes normally elicited by IFN–γ, including *Ifngr* (i.e., *Tnfa, Il1b, Cd54, Ifngr* etc.) (122, 210, 250).

Of note, the inhibition of macrophage activity is not always harmful. As demonstrated in a recent study, during *Mycobacterium ulcerans* infection causing Buruli ulcer disease, IFN–I response promotes macrophage tolerance, which facilitates spontaneous resolution of the necrotic inflammation (267).

Besides inhibiting macrophage reactivity to IFN–γ, IFN–I can modulate macrophage activity by restraining their metabolism. In *Mtb*–infected macrophages, IFN–β restrained glycolysis and drove mitochondrial stress via STING–dependent mechanism (268). During methicillin–resistant SA (MRSA) infection, IFN–I signaling disrupted oxidative phosphorylation through nitric oxide synthase (iNOS)–dependent mechanism (269).

#### 5.4.2 Induction of macrophage death

Macrophages can respond to a bacterial infection by dying through either programmed or non–programmed processes (202). IFN–I promote both types of macrophage death (117, 129, 270, 271). Theoretically, macrophage death may be either protective to the host due to the inhibition of the growth of intracellular pathogens, or deleterious due to the depletion of the macrophage population and increased dissemination of a pathogen. In most experimental settings, IFN–I–induced macrophage death was deleterious. During LM infection, IFN–I–dependent macrophage apoptosis depleted protective TNF–α–producing CD11b^+^ macrophages, which promoted disease severity (117). Increased resistance of *Ifnar1^–/–^* mice to ST infection was associated with a higher resistance of their macrophages to necroptosis (127, 129). Following *Francisella* infection, IFN–I response induced inflammasome–mediated macrophage death and macrophage depletion *in vivo* (78). IFN–I signaling triggered by SA upregulated the expression of proapoptotic genes and caspase–3 cleavage within macrophages and drove phagocytic cell apoptosis in the nasal tissue (189). IFN–I–dependent death of macrophages during experimental *Mtb* infection has recently been documented with the use of genome–wide CRISPR/Cas9 screening approach. The blockade of IFN–I signaling rescued macrophages and augmented the beneficial effect of rifampin. Unexpectedly, in this study, the mechanisms of macrophage death could not be attributed to any of already known pathways and they remain to be identified (158).

Overall, IFN–I largely inhibit macrophage activation, promote M2 polarization, and induce macrophage apoptosis. These effects contrast to the action of IFN–I on another myeloid population – DCs.

#### 5.4.3 Induction of trained immunity

Following exposure to various stimuli, macrophages can acquire memory–like features and the capacity to develop augmented responses to secondary challenges, features collectively referred to as trained immunity (TI). Mechanistically, TI is underpinned by epigenetic remodeling and metabolic reprogramming (272, 273). Classical TI inducers are molecules of microbial origin exerting strong and broad–spectrum activation of target cells, such as BCG, LPS or β–glucan. Emerging evidence indicates that IFN–I can also act as TI inducers: priming with IFN–β enhanced IL–6 production, altered cellular metabolism and lipid composition of mouse and human macrophages exposed to secondary stimuli (274). Furthermore, IFN–I appear to be a prerequisite for TI induction by BCG or LPS (275, 276). Importantly, trained immunity is not restricted to macrophages and has been described across multiple innate immune cell types, as well as in hematopoietic stem cells and non–hematopoietic cells (see Section 6.2).

Several questions related to the involvement of IFN–I in TI arise. First, various cytokines, such as TNF–α and IFN–γ, also induce TI. The complex relationships between these cytokines and IFN–I raise the question of how they interact during the formation of macrophage memory. Second, LPS while a classical TI inducer at low doses, can induce macrophage tolerance (i.e., attenuated responsiveness to secondary challenge) if applied repeatedly or for prolonged periods (277). This prompts further questions: can IFN–I be tolerogenic? If so, which factors determine their training *versus* tolerogenic potential? Supporting a tolerogenic potential of IFN–I, IFN–β–primed macrophages mounted a memory–like response to secondary challenge with LPS but showed reduced reactivity to Poly(I:C) (274). Another important question is whether IFN–I can induce TI *in vivo* and how this influences host anti–infectious protection. In the study by Lai and co–authors, BCG–primed human macrophages exhibited elevated antiviral activity *in vivo* due to increased IFN–I responses resulting from augmented cholesterol accumulation, a key feature of trained macrophages (276). Similarly, *in vivo* priming of pulmonary cells with i.n. administered LPS trained alveolar macrophages, enhanced their IFN–I responses, and mitigated subsequent pneumococcal infection (275). However, in the same study, adoptive transfer of LPS–primed alveolar macrophages to naïve recipients worsened the subsequent pneumococcal infection, leaving the question of the protective potential of trained macrophages open.

#### 5.4.4 Modulation of DC differentiation, activation and T–cell polarizing activity

DCs are key drivers of T–cell responses and important contributors to antibacterial immunity. They comprise several populations with distinct origins and functions. pDCs and cDCs develop in the BM from a common precursor. pDCs are the primary IFN–I producers, whereas cDCs specialize in antigen presentation, although they can also produce IFN–I (278–281). Monocyte–derived DCs (moDCs) arise from circulating monocytes. A subset of moDCs, TiP–DCs, was described during LM infection and shown to produce IFN–β (48, 282).

IFN–I effects on DCs have been studied predominantly in noninfectious settings and in viral and tumor models, with relatively few studies in bacterial infection contexts. The principal experimental model involves *in vitro* differentiation of monocytes and CD34^+^ progenitors into DCs using GM–CSF or GM–CSF and IL–4 (reviewed in (283)). In the absence of infectious stimuli, IFN–α/β generally accelerate DC differentiation and promote their proinflammatory phenotype characterized by TNF–α, IL–6, and IL–8 production, increased adhesion molecule expression, enhanced T helper cell–stimulatory capacity, and expression of NK–associated markers (284–286). Inhibitory effects of IFN–I on DC differentiation have been reported only in a limited number of studies (287). In bacterial contexts, however, IFN–I appear to impair DC differentiation: when added together with LPS or lipoteichoic acid, IFN–I exerted pro–apoptotic effects and reduced TNF–α and IL–12p70 production in surviving cells (288).

Recent work has revealed plasticity between pDCs and cDCs: in the presence of TNF–α, pDCs undergo reprogramming and acquire transcriptional, epigenetic, and functional features of cDCs, including antigen–presenting capacity. IFN–I were shown to inhibit pDC–to–cDC reprogramming, which may have implications for T–cell suppression (281). Whether such reprogramming occurs during bacterial infections, where IFN–I levels are often elevated, remains unknown.

To effectively stimulate T cells, DCs must undergo maturation. Maturation triggered by various stimuli, including microbial components, upregulates MHC and costimulatory receptors, enables antigen cross–presentation to CD8^+^ T cells, and promotes Th1 polarization. These activities are, however, transient and decline after initial DC activation (289). In *in vitro* models, IFN–I not only accelerated the maturation of immature committed DCs but rendered it more stable: IFN–I–exposed DCs sustained enhanced MHC II and costimulatory molecule expression, promoted T–cell proliferation and Th1 responses and upregulated chemokine receptors mediating lymph node homing (213, 284, 289–294).

The pattern of DC–derived cytokines ultimately determines T–cell polarization. IFN–I stimulate IL–12 production by DCs in some settings, but inhibit it and promote the production of IL–10 in others (209, 292). The outcome appears to depend on DC maturation state: IFN–β favored the formation of DCs promoting Th1 polarization when acting on immature DCs (a likely model of early infection stages), yet inhibited Th1–polarizing capacity in mature DCs (209). Duration of IFN–I exposure also matters: prolonged exposure to IFN–I promoted IL–10 and PD–L1 expressions (295). The response is further shaped by pathogen type: in the context of helminth infection, IFN–I promoted DCs to stimulate the development of Th2 cells (259). In bacterial settings, virulent *Francisella* suppressed DC secretion of IL–12p40 via IFN–β induction (296).

Overall, IFN–I generally stimulate DC differentiation and maturation. Th1 polarizing activity is supported by low dose and short–term IFN–I exposure (conditions characteristic for early infection stages) and inhibited by high doses or prolonged IFN–I action (chronic and/or severe infections). The stimulatory effects of IFN–I on DC differentiation and maturation contrast with the predominantly inhibitory effects of IFN–I on macrophage activation. Given the central role of DCs in initiating adaptive immune responses, these differences are likely biologically significant, although the underlying mechanisms, potentially involving distinct PRR expression and signaling, remain incompletely understood.

### 5.5 Neutrophils

Neutrophils are among the first cells to arrive at the infectious site. Their antibacterial activity is mediated via phagocytosis, degranulation, the generation of reactive oxygen species (ROS), the formation of neutrophil extracellular traps (NETs) as well as through the release of cytokines, chemokines and enzymes acting on other immune cells and propagating local immune reactions (297–299). The essential role of neutrophils in anti–bacterial defense is substantiated by a marked vulnerability of neutropenic patients to bacterial infections (300). However, the same mechanisms that mediate neutrophil–dependent defense can become detrimental if neutrophilic inflammation becomes chronic and gets out of the control. The pathological role of neutrophils is supported by multiple observations on a direct association between the degree of neutrophilic inflammation and the severity of certain bacterial infections, particularly, TB (153, 299, 301–303). IFN–I affect different aspects of neutrophil functionality.

#### 5.5.1 Netosis

IFN–I stimulate the formation of NETs. Paradoxically, IFN–I–stimulated NETs rather promote than repress bacterial growth. In a model of PA infection, *in vitro* treatment of neutrophils with recombinant IFN–β enhanced NETosis and stimulated the formation of PA biofilms. In *Ifnb*^−/−^ and *Ifnar1*^−/−^ mice, NETs formation was reduced, which, along with a reduced formation of ROS, accounted for a higher resistance of these mice to PA (187). In TB–susceptible C3HeB/Fe mice, IFN–I–induced NETosis promoted extracellular growth of *Mtb* and disease progression. Similar results were obtained in B6 mice rendered susceptible to *Mtb* through the treatment with anti–GM–CSF antibodies (304). Chowdhury and co–authors have recently demonstrated that during *Mtb* infection IFN–I promote the formation of chromatin–containing vesicles, this stimulates NET formation but simultaneously preserves the integrity of plasma membrane, neutrophil viability and inflammation (305). Of note, IFN–I and NETs form a positive regulatory loop, which propagates IFN–I/NETs deleterious effects: IFN–I stimulate NETosis, NETs contain nucleic acids able to trigger IFN–I (266, 306).

#### 5.5.2 Neutrophil death

Neutrophils have a relatively short life span in steady–state conditions and even more so during infections (298). IFN–I promote neutrophil death, which may have deleterious consequences due to a reduction in the first–line–defense population and via the induction of tissue necrotizing processes. Mechanisms reported as underlying IFN–I–dependent neutrophil death include: (i) repression of G–CSF, GM–CSF and other cytokines supporting neutrophil survival and (ii) stimulation of the production/expression of ROS and Fas, factors, implicated in neutrophil apoptosis and necrosis (307–309).

### 5.6 Myeloid cell recruitment to the infectious site

Migration of myeloid cells to the site of pathogen entry and persistence is a key event in host response to infection.

The recruitment of macrophages can be both stimulated and inhibited by IFN–I. An enhancement of macrophage recruitment was observed following LM and *Mtb* infections (118, 124, 152, 153), as well as in response to Poly(I:C), pristane–induced chronic peritonitis and viral infections (118, 124, 152, 310–313). In these models, deletion of *Ifnar1* attenuated macrophage accumulation, supporting that the processes depended on the integrity of IFN–I signaling. The main mechanism considered as being responsible for IFN–I–stimulated macrophage recruitment involves IFN–I–dependent upregulation of CCR2 on monocytes and stimulation of the local secretion of CCL2 (153, 312, 313). In other studies, however, deletion of *Ifnar1* augmented the accumulation of macrophages at the sites of infection. Specifically, this was observed following SPn, ST, as well as *Mtb* and LM infections (117, 127, 159, 184, 185).

The analysis of the relationships between IFN–I effects on macrophage recruitment and overall infection severity does not yield a consistent pattern. The pro–recruiting activity of IFN–I was associated with milder infection in some studies (e.g., LM infection (118, 124)), but correlated with a more severe disease course in others (e.g., *Mtb* infection (152, 153)). Similarly, IFN–I–dependent inhibition of macrophage recruitment was linked to IFN–I–mediated protection in some cases (SPn, *Mtb* (159, 184, 185), but not in others (LM (117)).

Data on the influence of IFN–I on the recruitment of neutrophils are even more confusing.

In some settings, IFN–I inhibited neutrophil recruitment, and this was detrimental for the host. Following *Francisella* infection, the absence of IFN–I signaling increased neutrophil influx to the spleen (136). *Ifnar1^–/–^* mice infected with influenza virus and challenged with SPn had improved neutrophil responses during the early phase of the bacterial infection and a better pathogen clearance as compared with WT mice (196). Similarly, *Ifnar1^–/–^* mice challenged systemically with LM exhibited an increased recruitment of neutrophils to the spleen and to the peritoneal cavity and a reduced local bacterial burden (54, 123). An intravital imaging of the livers of LM–challenged mice revealed a sustained local expression of IFN–β, which inhibited neutrophil swarming and hampered neutrophil–mediated eradication of the bacteria (120). In all of these studies, IFN–I–dependent inhibition of neutrophil recruitment was associated with an overall detrimental effect of IFN–I on the infection course.

Mechanistically, the inhibition of neutrophil influx has been attributed to IFN–dependent repression of the production of neutrophil–attracting chemokines, primarily, CXCL1 (KC) and CXCL2 (MIP–2), as well as the IL–17 cytokine. Thus, *in vitro*, exogenously added IFN–β suppressed CXCL1 and CXCL2 secretion by mouse and human macrophages (65). *In vivo*, knockout of *Ifnar1* enhanced CXCL1 and CXCL2 levels in mice challenged with LM, ST and SPn (65, 123, 196). In *Ifnar1^–/–^* mice co–infected with influenza virus and PA, an enhanced recruitment of neutrophils to the lungs was due to a higher expression of IL–17 by γδ T cells (314). Pathways suggested as being responsible for the IFN–I–dependent inhibition of CXCL1/CXCL2/IL17 axis include the augmented production of IL–10 (able to restrict *Cxcl1/Cxcl2/IL17* expression (315, 316)) and the induction of SET domain bifurcated 2 (Setdb2) lysine methyltransferase (198).

However, in other settings, particularly, during *Mtb* infection, IFN–I signaling promoted rather than inhibited the accumulation of neutrophils, and this was also detrimental for the host (151, 153). TB is a chronic infection that can be modeled in mice by a low–dose aerosol challenge with *Mtb* (317, 318). In mice of most strains, the challenge induces a long–lasting chronic infection. However, mice of some strains rapidly succumb to the infection (317). In such TB–susceptible mice, rapid disease progression has been associated with an excessive infiltration of the lung tissue with neutrophil–like cells (319–321). IFN–I appear to be directly involved in this process: TB–susceptible mice lacking IFNAR1 displayed a repressed secretion of CXCL1/CXCL5, a reduced recruitment of neutrophils to the lungs and were protected from death (153). In mice challenged with *M. bovis*, treatment with neutralizing anti–IFN–I antibodies reduced neutrophil recruitment to the lung, decreased bacterial burden, ameliorated lung pathology and prolonged survival (88). In a recent study by Saqib and co–authors, IFN–I drove the recruitment of a specific CD101^–negative^ pathology–associated subset of neutrophils in CXCR2–dependent manner (322). In patients, TB severity has also been linked to neutrophil abundance (303, 323).

In contrast to *Mtb* infection, in several other models, the capacity of IFN–I to augment neutrophilic inflammation was protective. The examples include ST infection (66, 132), intragastrically–induced LM infection (118), and a mouse model of a sequential sepsis → pneumonia infection (324). Paradoxically, in these models, the stimulatory effect of IFN–I on neutrophil recruitment has been attributed to the enhanced production of the same factors that in other models were inhibited by IFN–I, such as CXCL1 and IL–17. Finally, a number of studies did not detect any effect of IFN–I on neutrophil peripheral accumulation (54, 100, 114, 127, 152).

The inconsistency of the data cannot be explained only by the differences in pathogen biology since contradicting results have been obtained even following the infection with the same pathogen (illustrated in **Tables 1**-**5**). With this regard, one of the parameters that must be taken into account is how myeloid cells were detected in the studies. Indeed, to identify macrophages, different groups used different sets of markers, e.g., CD11b^+^F4/80^+^Gr1^int^ (152), F4/80^+^Gr–1^+^ (312), CD45^+^Gr1^+^CD11c^+^ (185), Ly6C^hi^Ly6G^–^ (118, 311) or CD11b^+^B220^–^Ly6C^+^Ly6G^–^ (310). This could lead to the analysis of somewhat different subsets of monocytes/macrophages. For example, the population of lung macrophages includes alveolar, inflammatory and resident macrophages, which differ phenotypically and are differentially influenced by IFN–I (152). Furthermore, phenotypically distinct subsets may vary in their maturation statues, which influences both their functionality and their role in protection or pathology. Thus, cells expressing Gr–1 or Ly–6G at high and intermediate levels have been identified as neutrophils and immature myeloid cells, respectively, and these populations differentially contributed to the pathogenesis of *Mtb* infection (320, 321).

In summary, IFN–I can stimulate, inhibit or have no effect on myeloid cell recruitment to the sites of bacterial infection, and the same effect may be either protective or detrimental. Most studies have linked IFN–I–dependent modulation of myeloid inflammation with alterations in the production of, and cell reactivity to, monocyte/macrophage– and granulocyte–attracting chemokines. However, the observed effects are difficult to explain solely in terms of cell trafficking, at least in the case of enhanced recruitment, as such recruitment requires that the cells be abundantly generated. This raises the question of whether, and if so how, hematopoiesis (HP), and specifically myelopoiesis, are affected by IFN–I.

## 6 IFN–I and myelopoiesis

### 6.1 The hierarchical scheme of adult hematopoiesis

**Figure 3:**
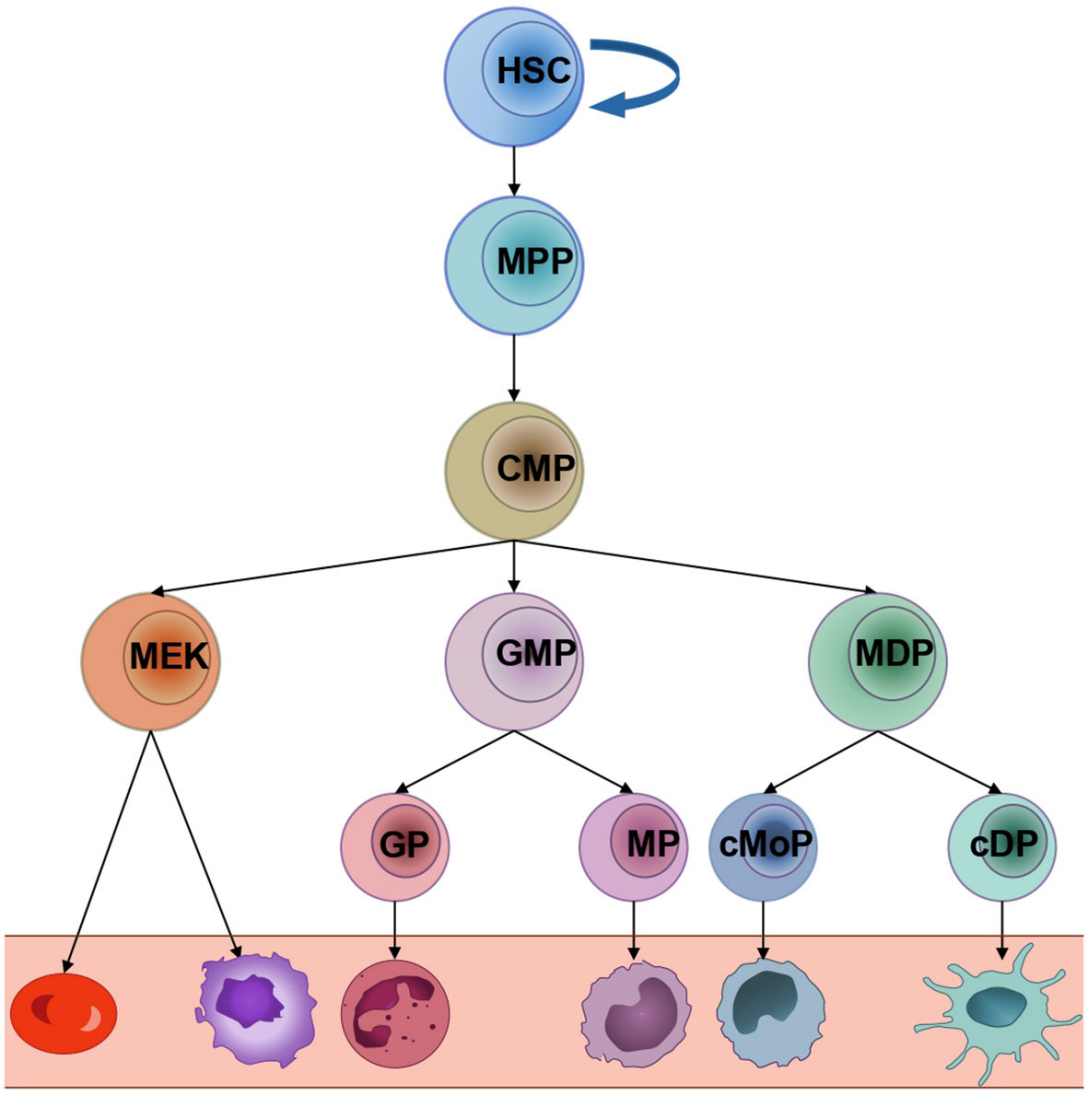
Adult myelopoiesis scheme. HSCs reside in the bone marrow in a quiescent state, periodically self–renew and differentiate into all types of mature blood cells on demand. The classical hematopoietic scheme implies that HSCs give rise to MPPs that differentiate into CMPs and CLPs. In myelopoiesis, CMPs give rise to MEKs, GMPs and MDPs. GMPs differentiate into GPs and MPs giving rise to monocytes and all types of granulocytes (273). MDPs form cMoPs and cDPs. Recent amendments to the classical hematopoietic scheme can be found in (275, 280–282). cDP, common dendritic cell progenitor; CLP, common lymphoid progenitor; cMoP, common monocyte progenitor; CMP, common myeloid progenitor; GMP, granulocyte–monocyte progenitor; GP, granulocyte progenitor; HSC, hematopoietic stem cell; MDP, monocyte–dendritic cell progenitor; MEK, megakaryocyte–erythrocyte progenitor; MP, monocyte progenitor.

In adults, HP takes place in the bone marrow in steady state conditions and can also occur extramedullary in pathological conditions. Hematopoietic stem cells (HSCs) form the basis of HP and are characterized by long–term quiescence, periodical self–renewal and the capacity to differentiate into all types of mature blood cells. According to the classical scheme of HP, when differentiating, HSCs first give rise to multipotent progenitors (MMPs), which together with HSCs, form the hematopoietic stem and progenitor cell population (HSPCs). MPPs engender cells of myeloid and lymphoid lineages. In the myeloid differentiation pathway, common myeloid progenitors (CMPs) are formed and give rise to megakaryocyte–erythrocyte (MEP), granulocyte–monocyte (GMP) and monocyte–dendritic (MDP) progenitors. GMPs differentiate into granulocyte (GPs) and monocyte (MPs) progenitors, while MDPs differentiate into common monocyte (cMoPs) and common dendritic cell (CDPs) progenitors (**Figure 3**) (325–328). Eventually, GPs, MPs, cMoPs and CDPs ensure the generation of monocytes, dendritic cells and all types of granulocytes. In steady–state conditions, HP is maintained by MPPs, which helps preserving the HSC pool. Under stress conditions, including infections, HSCs can start being involved in the HP (329–331). Although recent studies have revised the classical HP scheme (327, 332–334), its major principles remain valid, allowing us to use it when considering the hematopoietic effects of IFN–I.

### 6.2 IFN–I effects on HSPCs

The effects of IFN–I on HSPCs have mainly been studied by exposing mice or isolated HSPCs to exogenous IFN–I or poly(I:C). Such treatments make HSCs to exit quiescence and enter the cell cycle (335–338), promoting their differentiation rather than self–renewal (339). In various models, including prolonged Poly(I:C) or recombinant IFN–α exposure, sepsis and viral infections, inflammatory HSC cycling has been shown to deplete the HSC pool, impair long–term repopulation, and lead to BM failure and pancytopenia (338, 340–343). The details of these alterations differ between studies. Pietras and colleagues found that chronic exposure of mice to Poly(I:C) drove HSC division and led to BM aplasia, however, these alterations were transient, and HSCs returned to a quiescent state without losing their repopulating capacity (338). In contrast, *Ehrlichia* infection caused a marked reduction in HSC and HSPC pools due to IFN–I–dependent cell death rather than exhaustion from cycling (344). In the study by Zhang and colleagues (341), both sepsis induced by cecal ligation and puncture, as well as LPS administration, drove expansion of HSCs and HSPCs in a TRIF–dependent manner. More recently, Han and colleagues demonstrated that LPS–induced HSC cycling is exhausting and impairs HSC reconsitutive capacity (345). Across all studies, these effects were mediated by IFN–I.

Upon exposure to pathogens or pathogen–induced cytokines, HSPCs not only alter their proliferative activity but can also undergo long–lasting epigenetic and metabolic reprogramming, resulting in enhanced responses to secondary infections, a phenomenon termed central trained immunity (346, 347). HSPC training can be transmitted to myeloid progeny, thereby shaping downstream innate immune responses. For example, influenza–trained HSPCs conferred increased resistance to *M. avium* (and vice versa), while macrophages derived from BCG–trained HSPCs exhibited enhanced *Mtb* killing in an IFN–γ–dependent manner (348). In contrast, *Mtb*–trained HSPCs imprinted BMDMs with an impaired resistance upon re–challenge, an effect shown to be entirely dependent on IFN–I signaling (349). While trained immunity is not the primary focus of this review, these observations raise the possibility that IFN–I–mediated effects on HSPC training may contribute to the context–dependent beneficial or detrimental outcomes of IFN–I during bacterial infections. Further studies are required to clarify these mechanisms.

IFN–I affect HSPCs not only under pathological conditions, but also at steady–state. Recent studies have shown that commensal bacteria induce basal levels of IFN–I, and these levels are required to maintain HSPC pool (350–352).

Taken together, IFN–I can regulate the size and functionality of HSPC pools both under steady–state conditions and in surrogate models of bacterial infections, such as administration of LPS or Poly(I:C). However, whether and how HSPCs are affected during actual bacterial invasion and how these effects influence infection severity and chronicity remains largely unexplored.

### 6.3 IFN–I effects on myeloid progenitors

Bacterial infections create an increased demand for innate immune cells, which is met through emergency hematopoiesis (eHP). eHP is a stress response to infection that activates HSPCs, biases their differentiation towards the myeloid lineage, and accelerates migration of the newly generated cells to the periphery (353, 354). To ensure rapid replenishment of peripheral innate immune cells, eHP proceeds through a shunt pathway, resulting in the generation of not fully mature myeloid cells. Factors driving eHP include growth factors, pathogen– and inflammation–induced molecules and pro–inflammatory cytokines, including IFN–I (236, 353, 355).

Data on IFN–I effects on myeloid progenitors are limited, and most studies have not been performed in bacterial context. In mice, IFN–β deficiency reduced the frequencies of granulocytes/macrophages colony–forming units (GM–CFUs) and erythroid burst–forming units (E–BFUs) and lowered the numbers of circulating Gr–1^+^ and Mac–1^+^ cells (222). In contrast, overexpression of TLR7 increased GMP percentages. These GMPs expressed Sca–1, were more proliferative than classical GMPs, and overexpressed ISGs such as *Mx1, Oas1*, and *Isg15*, indicating that they arose as a result of IFN–I–induced eHP (356). An IFN–I–dependent expansion of GMPs was also observed during influenza A virus infection in mice (357). A detailed analysis of HP in the context of a real bacterial infection was carried out in a model of *Ehrlichia muris* infection. Differentially form other studies, the authors observed a significant reduction in GMP and CMP populations in infected mice. However, these alterations dependent on IFN-γ and were not mediated by IFNAR1 signaling (358).

An intriguing and still unanswered question is how IFN–I affect myelopoiesis downstream of progenitor cell populations. In clinical settings, most bacterial infections induce granulocytosis, however, certain infections, particularly chronic ones, induce monocytosis. Several studies have reported that IFN–I favor monopoiesis at the expense of granulopoiesis. In a model of influenza A virus infection, IFN–I increased the expression of M–CSF receptor on GMPs and MPs, which favored the generation of monocytes, reduced the generation of granulocytes and increased host susceptibility to secondary SPn infection (357). In a model of non–lethal endotoxemia, *E. coli* LPS increased monocytopoiesis and promoted the differentiation of bipotent MDPs into monocytic antigen–presenting cells in an IFN–I–dependent manner (359). Endotoxemia typically presents in two phases, an acute hyperinflammatory and a later immunosuppressive one. In a human *in vivo* model of LPS–induced systemic inflammation, the immunosuppressive phase was characterized by diminished expression of IFN–I–responsive genes in monocytes and impaired myelopoiesis. Treatment with IFN–β restored both IFN–I responses and monocyte maturation (360).

Other studies link IFN–I signaling to granulocytic response. In progressing TB, neutrophils and immature granulocytic myeloid cells accumulate in the periphery and BM (303, 319–321, 361) and express IFN–I–inducible transcripts, indicating that they or their progenitors have been exposed to IFN–I (86). A direct demonstration of IFN–I ability to promote granulopoiesis has recently been obtained by single–cell analysis of BM cells derived from patients with myeloproliferative neoplasms undergoing IFN–α treatment (362). The treatment induced the appearance of inflammatory granulocyte progenitors (IGPs) that expressed granulocyte–associated markers and transcriptional factors (such as CD66b, *CEBPB*, *CEBPD*) and IFN–I–inducible genes (*IRF1* etc.) and were transcriptionally similar to quiescent HSCs, which allowed the authors to suggest that IGPs differentiated directly from HSCs, bypassing intermediate differentiating stages, a trait characteristic of eHP.

In summary, there are currently only a limited number of studies examining hematopoietic effects of IFN–I. Most available data have been obtained either in non–bacterial settings or using surrogate models of bacterial infection, such as LPS treatment; the results are sometimes conflicting, especially regarding preferential promotion of mono– or granulopoiesis. However, the ability of IFN–I to interfere with hematopoetic processes is undeniable; it may determine the effects of IFN–I on innate immune responses, disease severity and outcomes. Understanding hematopoietic effects of IFN–I and mechanisms underlying them could help in developing new strategies to treat severe life–threatening infections.

## 7 Conclusions and perspectives

Taken together, the studies reviewed here highlight the dual and context–dependent role of IFN–I in bacterial infections, bridging fundamental immunology with potential clinical applications.

### 7.1 Context–dependent roles of IFN–I

IFN–I play complex, context–dependent roles during bacterial infections, contributing to both host protection and immunopathology. Their effects vary with pathogen species, infection stage, route of infection, tissue niche and host immune status, reflecting the dual nature of IFN–I signaling in antibacterial defense.

### 7.2 Beyond classical cytokine modulation: impact on myeloid cells

Most studies on IFN–I have focused on classical immune pathways, including proinflammatory cytokines, IL–10, and T–cell responses, and reported inconsistent results. In contrast, the effects of IFN–I on the myeloid compartment remain underappreciated. IFN–I can influence recruitment and survival of neutrophils and monocytes, and importantly, their generation during emergency HP. A major knowledge gap remains in understanding how IFN–I signaling orchestrates HP and myelopoiesis during bacterial infections. Dysregulation of these processes may underlie severe, progressive infections and represents both a mechanism of disease progression and a potential therapeutic target.

### 7.3 Clinical applications

#### 7.3.1 IFN–I as biomarkers

IFN–I–driven transcriptional signatures have emerged as informative biomarkers in several bacterial diseases, such as tuberculosis and sepsis. IFN–I responses during viral infections influence susceptibility to secondary bacterial infections. Thus, assessment of IFN–I activity, e.g., via ISG signatures, may aid early risk stratification, diagnosis and monitoring of disease dynamics.

#### 7.3.2 Therapeutic opportunities

In selected extracellular bacterial infections where IFN–I are protective, therapeutic augmentation of IFN–I responses could be considered, particularly, for antibiotic–resistant pathogens. Conversely, the detrimental role of IFN–I in chronic intracellular or late–stage infections suggests that transient, targeted inhibition may be beneficial, especially in severe disease with profound immune dysregulation. Dysregulation of myelopoiesis may serve as a biomarker indicating the need for such therapy.

#### 7.3.3 Limitations

Clinical translation of IFN–I–based approaches is constrained by several factors. First, IFN–I effects are highly context–dependent, and the determinants of protective versus pathological responses remain poorly understood. Second, robust quantitative data on circulating IFN–α/β in bacterial infections are limited, most evidence comes from blood transcriptional signatures or animal/ex vivo studies. Third, systemic IFN–I administration carries risks of adverse effects, as observed in oncology settings (363). Finally, patient stratification and monitoring tools for safe IFN–I modulation are lacking. These limitations currently preclude IFN–I therapeutic use and highlight the need for additional mechanistic studies and carefully designed translational trials.

### 7.4 Perspective and future directions

Important objectives for future work include:

- Integrating mechanistic insights on IFN–I–mediated myeloid regulation with clinical observations.
- Clarifying determinants of protective versus pathological IFN–I responses.
- Identifying patient populations likely to benefit from IFN–I modulation.
- Developing strategies to safely harness or inhibit IFN–I in bacterial infections.

## Abbreviations

AP–1: activator protein 1
BCG: Bacille Calmette–Guérin
BMDMs: bone marrow–derived macrophages
c–di–AMP: cyclic diadenosine monophosphate
cDCs: conventional dendritic cells
cGAS: cyclic GMP–AMP synthase
CMP: common myeloid progenitors
DCs: dendritic cells
dsRNA: double–stranded RNA
eHP: emergency hematopoiesis
FAK: focal adhesion kinase
FT: *Francisella tularensis*
GAS: interferon–gamma–activated sequence
GMP: granulocyte–monocyte progenitor
GPs: granulocyte progenitors
HSC: hematopoietic stem cells
HSPC: hematopoietic stem and progenitor
i.n.: intranasal
i.p.: intraperitoneal
i.v.: intravenous
IFNAR: Interferon–alpha/beta receptor
IFN–I: type I interferons
IKKϵ: inhibitor of κB (IκB) kinase–related kinase
IRAK: Interleukin–1 Receptor–Associated Kinase
IRF: interferon regulatory factor
ISG: interferon–stimulated gene
ISGF3: Interferon Stimulated Gene Factor 3
ISRE: interferon–stimulated response elements
KP: *Klebsiella pneumonia*
LCMV: lymphocytic choriomeningitis virus
LM: *Listeria monocytogenes*
LPS: Lipopolysaccharides
LVS: *F. tularemia* live vaccine strain
MAPK: mitogen–activated protein kinase
MAVS: mitochondrial antiviral signaling protein
MDA–5: melanoma differentiation–associated gene 5
MDP: monocyte–dendritic cell progenitor
MEP: megakaryocyte–erythrocyte progenitor
MPP: multipotent progenitor
MPs: monocyte progenitors
*Mtb*: *Mycobacterium tuberculosis*
MyD88: Myeloid differentiation primary response 88
NETs: neutrophil extracellular traps
NF–κB: nuclear factor kappa–light–chain–enhancer of activated B cells
NOD1/NOD2: nucleotide–binding oligomerization domain–containing proteins
PA: *Pseudomonas aeruginosa*
pDCs: plasmocytoid dendritic cells
Poly(I:C): polyinosinic:polycytidylic acid
PRRs: pattern recognition receptors
RIG–I: retinoic acid–inducible gene I
ROS: reactive oxygen species
*S. Pyogenes*: *Streptococcus pyogenes*
SA: *Staphylococcus aureus*
SCV: *Salmonella*–containing vacuole
SPn: *Streptococcus pneumonia*
ss RNA: single–stranded RNA
Sst1: S*uper susceptibility to tuberculosis 1*
ST: *Salmonella enterica* serovar *Typhimurium*
STAT: Signal Transducer and Activator of Transcription
STING: stimulator of interferon genes
TAK1: Transforming Growth Factor Beta Activated Kinase 1
TB: tuberculosis
TBK1: TANK–binding kinase 1
TI: trained immunity
TLR: toll–like receptor
*Tpl2*: tumor progression locus 2
TRAF: Tumor Necrosis Factor Receptor–Associated Factor
TRIF: Toll/interleukin–1 receptor–containing adaptor molecule
WT: wild type.
